# How can social insurers promote return to work in occupational rehabilitation? A quantitative, cross-sectional study

**DOI:** 10.1186/s12889-021-11758-w

**Published:** 2021-09-16

**Authors:** Jarna Pasanen, Arto Luoma

**Affiliations:** Faculty of Management and Business, Tampere University, FI-33014 Tampere, Finland

**Keywords:** Occupational rehabilitation, Return to work, Insurers, Disability insurance

## Abstract

**Background:**

Earlier studies indicate a correlation between disability claims experience and return to work outcomes. Thus, the insurer’s role and actions may affect the self-rated health of the disabled worker and the outcomes of occupational rehabilitation. This study diversifies the existing empirical evidence on the role of the insurer in the rehabilitation process and reveals the critical actions that best promote success.

**Materials and methods:**

Explorative factor analysis (EFA) and confirmatory factor analysis (CFA), followed by binary regression, were used to analyse survey data of disabled workers (*n* = 661) who had undergone an occupational rehabilitation within an earnings-related pension insurance system in Finland.

**Results:**

The claimant’s perceptions of the insurer’s (1) high-quality informing and guidance, (2) customer orientation, (3) smooth process flow and (4) positive service attitude had substantial and statistically significant effects on the success of occupational rehabilitation after adjusting for all likely confounding variables.

**Conclusions:**

The insurer’s actions are significant predictors of the outcome of occupational rehabilitation. The insurer can promote the health of rehabilitees most effectively by ensuring a smooth process flow and adopting a customer-oriented approach.

**Supplementary Information:**

The online version contains supplementary material available at 10.1186/s12889-021-11758-w.

## Background

In most developed countries, public compensation systems providing support for those unable to work because of illness or injury are usually governed by public institutions or private insurance providers. Many disability insurance systems only cover injuries or illnesses if they are due to a specific cause, including workers’ compensation, automobile insurance and crime victim’s insurance. These systems are referred to as cause-based systems. However, there are also disability-based systems that provide sickness and disability insurance for all forms of work disability, regardless of the cause [1].

While there are huge disparities between and within nations when it comes to public disability insurance systems, they still have common features regarding the compensation process and the role of the administrative body (whether it is a public social insurance agency or private insurance company). This organisation is usually responsible for the compensations and it interacts with all the stakeholders involved in the rehabilitation and return to work process [1–3].

Today, the return to work (RTW) process after an illness or injury is understood as a biopsychosocial phenomenon [4]. This means that biological, psychological and social factors related to the personal, work, healthcare and insurance domains influence the course and outcome of rehabilitation [5]. Thus, the role and actions of rehabilitation stakeholders are seen vital to the outcomes of the process. Occupational rehabilitation stakeholders are usually categorized from system theory perspective into three main groups: employers (work system), healthcare providers (health system) and insurance (insurance system). Previous studies suggest that these stakeholders’ actions may affect the outcomes of the rehabilitation [6–8]. Our study focuses on the insurer’s actions (more specifically guidance, customer-orientation, process flow and service attitude among the insurer’s representatives) and their connection to successful rehabilitation.

Current theories suggest that more positive relationships between the insurer and claimants may improve the worker’s chance of recovery [9, 10] and vice versa. Qualitative studies focusing on relationships between disabled workers and healthcare or insurance officials highlight the importance of mutual trust, respect, and credibility [11, 12]. Furthermore, high-quality informing, professional expertise, a supportive and individual approach, a personal relationship with officials, participation in decision-making and individually chosen rehabilitation measures and goals are mentioned as promoters of RTW [12–15]. Quantitative studies [16–21] support the hypothesis of a causal relationship between insurer actions and the outcomes of occupational rehabilitation. Within a worker’s compensation system, the high stress levels from encounters with insurers were positively correlated with poor long-term recovery of injured workers [18] whereas a positive claims experience was strongly associated with having returned to work [3].

The results are similar between sick-listed individuals and social insurance officials in disability-based insurance schemes. Negative encounters with social insurance officers are negatively correlated with self-rated health, self-rated ability to work and mood, whereas positive encounters with social insurers facilitate RTW [17, 19–21].

As shown above, there is some evidence indicating a connection between insurer actions and the probability of RTW. However, there is a need for further evidence and a more comprehensive understanding of the insurer’s role and the specific actions that best support the health of disabled workers and promote RTW. While the earlier quantitative studies have concentrated on sick-listed patients or work injury victims, this article focuses on occupational rehabilitees, thus enlarging the scope and providing useful information to all rehabilitation stakeholders. Here, we do not collapse groups of stakeholders together (as e.g. in [16, 22]), but only examine the role of the insurer in order to eliminate the confounding factors related to other stakeholders. Our survey data enable a definition of dimensions related to the insurer’s role and their comparison with respect to RTW outcomes. The aims of this study are (1) to investigate the connection between insurer actions and the results of occupational rehabilitation, and (2) to explore which insurer actions best promote occupational rehabilitation success. Our research hypotheses, from the weakest (or least specific) to the strongest (most specific), are as follows:

- H1: Claimant’s perceptions of insurer actions during occupational rehabilitation are related to the outcome of rehabilitation.
- H2: Claimant’s perceptions of positive insurer actions, including high-quality informing, supportive service attitude and individual approach, are positively related to successful occupational rehabilitation.
- H3: The positive relationship between the claimant’s perceptions of positive insurer actions and successful occupational rehabilitation remains even if an adjustment is made for all likely confounding variables.

## Methods

This section presents the key elements of the study design, describes the research setting and participants, defines the used variables, their handling and measurement and presents the statistical methods. Here, we follow the STROBE statement [23].

### Study design

We used a cross-sectional survey design to investigate the connection between the claimant’s perceptions of insurer actions and the outcome of occupational rehabilitation. The study population included all private sector wage earners^1^ whose occupational rehabilitation process involved a service provider and whose rehabilitation case was closed in 2015. The rehabilitee’s case is closed in the online system when there is no further need for a service provider in the rehabilitation process. The main reason for this is that the rehabilitation assessments, plans for rehabilitation measures and all preparations needed to start the rehabilitation (e.g. finding an internship or student place) have been made. However, the rehabilitation measures themselves (such as retraining for an occupation) and the contact with the insurer often continue after this stage. Another reason for a closed case is the cancellation of occupational rehabilitation due to any reason, such as significant deterioration of the disability or other change in rehabilitation conditions.

The cross-sectional data were collected in 2017, about 2 years after the claimant’s rehabilitation case had been closed in the web service. This point of time was chosen because the occupational rehabilitation would probably be over by then for most claimants, and its outcome would be known. On the other hand, not too much time would have passed after the rehabilitation so that the claimants could still remember the insurer’s actions during the process.

### Setting and participants

The study sample was obtained from KuntoutuNET, a Finnish online service for the processing and maintenance of customer data about occupational rehabilitation. This service is used as a communication tool between the subscribers (all private sector pension institutions) and producers of rehabilitation services (e.g. institutions, companies, communities, and health-care units providing vocational rehabilitation services). The insurers may choose to use service providers to assist in the planning and execution of occupational rehabilitation within an earnings-related pension scheme in Finland. According to the Service Network of Occupational Rehabilitation (2016), service providers are used to assist in the settling and planning of occupational rehabilitation when the application for rehabilitation compensation lacks a rehabilitation plan, employment or work trials in a former or current workplace are not possible or when the rehabilitee needs close guidance. The service providers are also used to assess the rehabilitee’s abilities and opportunities for employment or studying [25].

The sample consisted of 2264 individuals and included all rehabilitees whose cases were closed in the KuntoutuNET online service in 2015. The data acquired from KuntoutuNET included the rehabilitee’s identification number, age, gender, and contact details (phone number and/or email-address). An online questionnaire was sent to the respondents on June 6, 2017, and reminder messages were delivered on June 13 and June 20, 2017. The data were collected using a text message and an e-mail survey. The survey was sent by e-mail to a total of 836 people whose e-mail addresses were stored in the KuntoutuNET online service. In addition, the survey was sent by text message to 1428 people, for whom only a telephone number was available.

### Variables and measurement

We took into account earlier empirical findings and considered potential effects of insurer actions on RTW outcomes when designing the questionnaire. In addition to questions related to sociodemographic variables and the process and outcomes of occupational rehabilitation, the questionnaire included altogether 26 propositions concerning the actions of the insurers and rehabilitation service providers. These propositions were formed on the basis of earlier studies [3, 7, 12–22], and the key terms were translated from English to Finnish by the authors. The questions were answered on a 5-point Likert scale with responses ranging from 1 (strongly disagree) to 5 (strongly agree). The sixth alternative, “doesn’t concern me” was coded as a missing value. Since our focus was on the actions of the insurer, we excluded from the analysis the questions purely related to the actions of rehabilitation service providers and ended up with 17 insurer-related propositions (listed as part of Table 4 in the Results section).

To isolate the effect of insurer actions, we included several potential confounding factors in our regression models, for example age, employment situation before rehabilitation, level of education, occupation, monthly net income and cause of rehabilitation. Age was included as a continuous variable and employment situation before rehabilitation as a dichotomic variable. The level of education was grouped into six categories: primary school, secondary school, vocational college, general upper secondary school, and university. The occupation had also six categories: employee, lower official, senior officer, leading position, entrepreneur, and agricultural entrepreneur. The cause of rehabilitation had three categories: musculoskeletal diseases, mental disorders and other causes; and monthly net income had six categories: below 500 euros, 500–999 euros, 1000–1499 euros, 1500–2499 euros, 2500–3999 euros, over 4000 euros.

Furthermore, we included the respondent’s motivation and the objectives of rehabilitation as possible confounding factors in our regression models. Motivation was measured on a 5-point Likert scale, the response alternatives ranging from 1 (not at all motivated) to 5 (extremely motivated). In addition, the questionnaire included six pre-formulated objectives for occupational rehabilitation: (1) return to work, (2) more suitable profession, (3) new job in earlier workplace, (4) improvement of professional capabilities, (5) improvement of mental well-being and (6) receiving a disability pension. The respondents were once more asked to use a 5-point Likert scale ranging from 1 (not at all important) to 5 (extremely important) to rate how important these objectives were to them before starting the process. Similar classifications regarding the motivation and objectives of rehabilitation have been used, for example, by Gould et al. [26].

The most frequently used measures of rehabilitation outcomes are self-rated health or self-rated ability to return to work (e.g. [17, 19, 20]) and employment status after rehabilitation (return to work) (e.g. [27, 28]). Here, we were able to use both measures. The respondents self-assessed their ability to work at the time of answering on a scale of 4 (totally unable to work) to 10 (excellent), which corresponds to the Finnish school rating scale and was therefore considered to be easily understood.^2^ In addition, the respondents were asked whether they had returned to work after the rehabilitation or not.

The employment status after rehabilitation was not an ideal measure of rehabilitation success in our study, since the RTW rate was only 30% and a significant amount of respondents were still in rehabilitation (for example, education leading to a degree may take several years to complete) or unemployed. Therefore, we formed a binary indicator of rehabilitation success by combining the self-rated ability to work and return to work variables. The rehabilitation was regarded as successful if the respondent had fully returned to work successfully or if the self-rated ability to work was 7 (satisfactory) or higher. We considered the objective of occupational rehabilitation (to improve the chances of a disabled individual to earn income in the future and to prevent permanent disability) to have been achieved in these situations.

### Statistical methods

Since our aim was to explore the connections between insurer actions and successful rehabilitation, we decided to use factor analysis to determine the dimensions of the insurer’s role, and binary regression to explain the result of rehabilitation by factor scores. There were 661 observations which had responses in the relevant variables, and in 108 cases some of the variables had missing values. To include these partially missing observations, we used the full information maximum likelihood (FIML) method.

The factor analysis was conducted in two steps: First, exploratory factor analysis (EFA) was used to determine the constructs or factors which best explain the observed correlations structure of the indicator variables, and, second, confirmatory factor analysis (CFA) was used to check the adequacy of the adopted factor model.

In the explorative analysis we used principal-axis factoring (PAF) since the data were not normally distributed. According to Fabrigar, Wegener, MacCallum and Strahan [29], the principal factor methods are the best choice if the assumption of multivariate normality is “severely violated” [30]. In our case, the responses were measured with the Likert scale, and had high means leading to negative skewness. However, the skewness went beyond the recommended limits of − 1.00 or 1.00 [30] only for two variables (PF1 and SA1; see Table 3), and even for them it was not very high (> 3).

Further, since the sample size was fairly large (549 observations after listwise deletion of missing data) and the results of EFA were almost identical to those obtained by the maximum likelihood estimation (MLE) method, we are confident that the results of CFA, which are based on the assumption of multivariate normality, are sufficiently accurate. Further, since the factors were obviously correlated, we used the promax rotation, which allows for such correlation. For EFA, we provide the factor loadings, means, standard deviations, Cronbach’s alphas, and average variance extracted (AVE) and construct reliability (CR) statistics for each measured item in Table 3.

In CFA, we first fitted a model corresponding to that from EFA. The resulting factor scores were used as independent variables in a logistic regression model explaining the success of rehabilitation. However, since the factor effects were ambiguous due to high factor correlation, we fitted another confirmatory factor model with independent factors (labeled as from F_1_ to F_4_) in order to separate the effects of the factors and to have a parsimonious correlation structure.

This model was not very well-fitting, so we added one further factor (labeled as G) to account for the correlations between the variable groups representing the independent factors. Factor G would represent the individual effect of the respondents, that is, their tendency to answer in a similar way to all questions. The factor loadings of G on all indicators were set to be equal. Further, since factor F_4_ had only two indicators, its loadings were also set equal to each other to make the model identifiable.

**Fig. 1:**
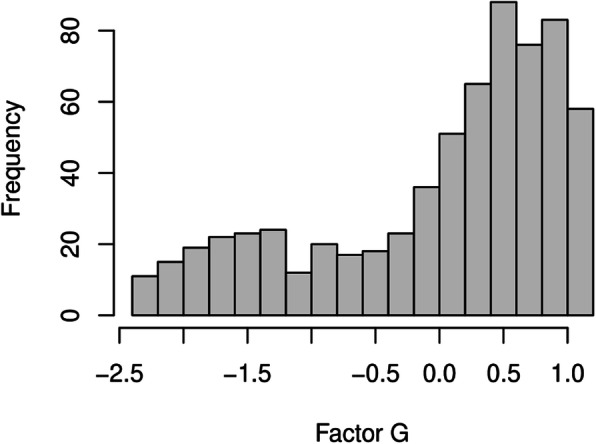
Histogram of the scores of factor G

In the next step, factor scores were computed using the regression method. It turned out that the distribution of the scores of factor G had a long-left tail (Fig. 1). However, this should not pose a problem since the regression method is based on the correlation structure of the variables and does not require the indicators or the factors to be normally distributed. Finally, logistic regression was used to regress the response variable on the factor scores. Probit regression yielded comparable results, but the fit of the logistic model was slightly better.

We also studied whether the connection between the insurer’s actions and the outcome of occupational rehabilitation could be explained by confounding variables. We added several likely confounding factors in our logistic regression model, including gender, age, level of education, employment situation, occupation, monthly net income, cause of rehabilitation, motivation and rehabilitation objectives. The demographics were computed and the explorative factor analyses conducted using the SPSS Version 26.0 for Windows; the CFA was run using the package lavaan (Rosseel, 2012) of the R software (R Core Team, 2020), and the logistic regression using the R function glm.

## Results

The response rate of the survey was 29% (*n* = 661). Table 1 shows that the proportion of respondents was higher in women than in men and slightly higher in younger people than in elderly.

**Table 1:** Descriptives of the respondents in relation to the intended sample

	Respondents (n)	Intended sample (n)	Response rate by gender and age (%)
**Gender**			
female	314	898	35%
male	347	1366	25%
**Age**			
25–34	62	201	31%
35–44	177	538	33%
45–54	257	881	29%
55–63	165	644	26%

Table 2 displays the basic characteristics of the respondents. The age range was 25–63 years, with a median age of 48. Most respondents were male (52%), had a vocational qualification (68%) and suffered from musculoskeletal diseases (66%). With the binary indicator of rehabilitation success (combining the self-rated ability to work and return to work variables), occupational rehabilitation proved successful for 60% (*n* = 395) of the respondents.

**Table 2:** Basic characteristics of the respondents

	%
**Gender**	
female	48,0
male	52,0
**Age**	
25–34	9,3
35–44	27,0
45–54	39,0
55–63	24,7
**Level of education**	
primary school	2,4
secondary school	13,4
vocational college	68,4
general upper secondary school	6,8
university of applied sciences	3,8
university	5,2
**Monthly net income (€)**	
0–499	1,2
500–999	13,4
1000–1499	23,7
1500–2499	44,5
2500–3999	14,5
4000-	2,6
**Cause of rehabilitation**	
Musculoskeletal diseases	66,4
Mental disorders	13,4
Other cause	20,2
**Life situation at the time of the survey**	
Rehabilitation ongoing	15,2
Successful RTW	30,1
Unemployed	20,9
Unable to work	27,1
Other situation	6,7
**Self-rated ability to work at the time of the survey**	
Poor (grade 4)	18,9
Tolerable (grades 5 to 6)	23,7
Good (grades 7 to 8)	41,4
Very good (grades 9 to 10)	16,0
**Rehabilitation outcome (binary variable)**	
Success	59,6
Failure	40,4

We conducted an explorative factor analysis (EFA) with four common factors for the 17 insurer related propositions. All these items exhibited sufficient factor loadings and communalities (> .50) and were retained for further analysis. The four factors accounted for 93.37% of the total variance, and each factor explained at least 4.34% of the total variance, fulfilling the minimal requirements presented by Netemeyer et al. [31]. Table 3 shows the estimated factor loadings, means and standard deviations of each measured item. Further, Cronbach’s alphas, and average variance extracted (AVE) and construct reliability (CR) statistics are provided for each factor. The four dimensions of the insurer’s role, their measures in the questionnaire and item codes are shown in Table 4. The propositions in Table 4 were translated from Finnish by the authors; these survey questions can be found in the original language in the appendix.

**Table 3:** Results of EFA

Item	Mean	Standard deviation	Crohn-bach’s alpha	Factor 1	Factor 2	Factor 3	Factor 4	CR	AVE
IG1	3.54	1.38	0.983	0.912				[.969]	[.842]
IG2	3.46	1.36		0.974					
IG3	3.71	1.35		0.910					
IG4	3.70	1.33		0.876					
IG5	3.64	1.33		0.914					
IG6	3.50	1.35		0.917					
CO1	3.39	1.44	0.984		0.892			[.972]	[.878]
CO2	3.72	1.31			0.926				
CO3	3.66	1.37			0.969				
CO4	3.59	1.35			0.967				
CO5	3.70	1.35			0.929				
PF1	4.01	1.27	0.972			0.894		[.943]	[.807]
PF2	3.85	1.23				0.933			
PF3	3.81	1.35				0.927			
PF4	3.78	1.26				0.835			
SA1	4.04	1.20	0.953				0.973	[.907]	[.832]
SA2	3.49	1.32					0.847

**Table 4:** Dimensions of the insurer role and their measures in the questionnaire

Insurer role -dimensions	Measure	Item code
Informing and guidance	I received sufficient information from the insurer	IG1
	The insurer’s instructions, announcements and information were clear and understandable	IG2
	I received expert service from the insurer	IG3
	The insurer’s staff were easily accessible	IG4
	The different rehabilitation options were adequately discussed	IG5
	I received sufficient information at all stages of the rehabilitation process	IG6
Customer orientation	The insurer considered my personal situation and individual needs	CO1
	The return to work plan reflected my own views and aspirations	CO2
	The return to work plan was feasible	CO3
	The content of the rehabilitation met my needs	CO4
	I had the opportunity to influence the rehabilitation process	CO5
Process flow	I was satisfied with the insurer’s compensation decision regarding rehabilitation	PF1
	The insurer started the settlement of rehabilitation at an appropriate time.	PF2
	The insurer carried out the rehabilitation measures quickly enough	PF3
	The rehabilitation process proceeded on schedule	PF4
Service attitude	The insurer’s customer service was friendly	SA1
	The insurer sought to highlight the positive aspects and opportunities	SA2

The first factor is theorized to represent informing and guidance during the rehabilitation process (variables IG1–IG6), the second factor is related to customer oriented approach (variables CO1–CO5), the third factor describes the process flow (variables PF1– PF4), and the fourth factor indicates the service attitude of the insurance personnel (variables SA1–SA2). Altogether, the sizes of the factor loadings (all above .8), AVE measurements (all above .8), and CR scores (all above .9) lend support to the convergent validity of each scale.

Next, we conducted a confirmatory factor analysis (CFA) based on the results above. We first fitted a model with a free factor correlation structure and zero restrictions on factor loadings as indicated by EFA. The model fit was good (CFI = 0.990, SRMR = 0.014, RMSEA = 0.046) although the hypothesis of perfect fit was rejected (χ^2^ = 310.8, df = 130, *p* = .000). When the resulting factor scores were used as independent variables in a logistic regression model explaining the success of rehabilitation, we could find support for hypothesis H_1_, which states that the claimant’s perceptions of insurer actions during occupational rehabilitation are related to the outcome of rehabilitation.

However, since it was not possible to separate the effects of the factors due to high factor correlations (ranging from 0.548 to 0.746), we fitted another model with uncorrelated factors. The model had an inferior and inadequate fit (χ^2^ = 1823.5, df = 137, *p* = .000; CFI = 0.906, SRMR = 0.496, RMSEA = 0.136). Finally, we fitted a factor model, with four independent factors as indicated by EFA, and one further independent factor accounting for the individual effect and correlation between the variable groups. The fit was almost as good as in the first case (χ^2^ = 390.2, df = 136, *p* = .000; CFI = 0.986; SRMR = 0.060; RMSEA = 0.053). The items’ squared multiple correlations (SMCs) ranged from 0.853 to 0.945.

**Fig. 2:**
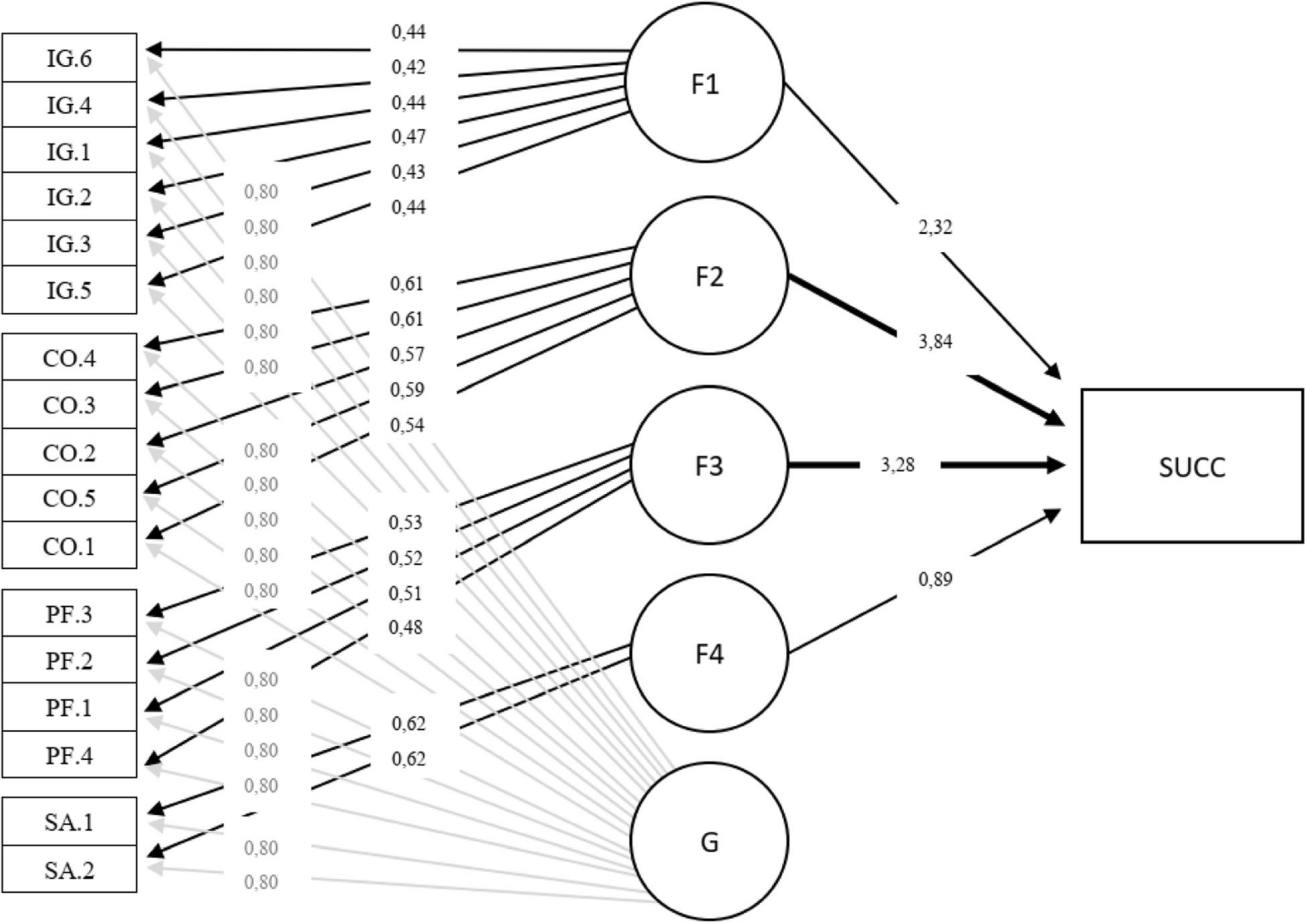
Results of CFA and logistic regression combined

Then, factor scores were computed using the regression method. These scores, excluding the individual effect score, were used as independent variables in a logistic regression model explaining the success of rehabilitation. Figure 2 shows the path coefficients for all the relationships in our two-stage model. All coefficients are statistically significant at the 0.001 level. The coefficients of the factor measurement model are standardized. The results suggest that the insurer’s customer oriented approach (β = 3.84) and smooth process flow (β = 3.27) have the highest impact on the success of occupational rehabilitation, but also proper informing and guidance (β = 2.32) and positive service attitude (β = 0.89) are associated with positive rehabilitation outcomes. The pseudo-R^2^ values (McFadden = 0.503, Cox and Snell = 0.490, Nagelkerke = 0.664) all indicate excellent model fit. Obviously, this result supports our research hypothesis H_2_, which states that the claimant’s perceptions of positive insurer actions are positively related to successful occupational rehabilitation.

We also studied whether these results could be explained by confounding variables (gender, age, level of education, employment situation, occupation, monthly net income, cause of rehabilitation, motivation and rehabilitation objectives), which are background variables affecting both the independent variables and the outcome variable. We found that age, self-rated ability to work before rehabilitation, motivation, and the objectives of either professional development or receiving disability pension, along with the insurer factors, were statistically significant predictors of the rehabilitation outcome. Table 5 shows that the inclusion of these explanatory variables slightly decreased the effect estimates of the insurer factors but did not render them statistically insignificant. The pseudo-R^2^ values improved (McFadden = 0.579, Cox and Snell = 0.538, Nagelkerke = 0.730). The number of included observations was 608. Thus, we find support for the research hypothesis H_3_, according to which the positive relationship between the claimant’s perceptions of positive insurer actions and successful occupational rehabilitation remains even if an adjustment is made for all likely confounding variables.

**Table 5:** Logistic regression model for the successful rehabilitation outcome

Explanatory variable or factor	Coefficient	z-value
F1 - Informing and guidance	1.96***	6.03
F2 - Customer orientation	3.46***	9.85
F3 - Process flow	3.10***	8.87
F4 - Service attitude	0.654*	2.36
Age	−0.042*	−2.34
SRH before rehabilitation	0.258*	2.41
Motivation	0.405*	2.19
Objective: professional development	0.399**	2.93
Objective: receiving disability pension	−0.355***	−3.60

Signif. Codes: 0 ‘***’ 0.001 ‘**’ 0.01 ‘*’ 0.05 ‘.’ 0.1 “1

## Discussion

Our purpose was to explore the connection between the insurer’s actions and the outcome of occupational rehabilitation. Factor scores measuring the dimensions of the insurer’s role were used as independent variables in a binary regression model explaining the success of rehabilitation. According to earlier studies, positive encounters with insurers improve the worker’s chance of recovery after an illness or injury and vice versa [9, 10]. Our results confirm these earlier perceptions and enlarge evidence-based understanding of the effects of insurer actions on the outcomes of occupational rehabilitation. This study contributes to the literature by identifying the different dimensions of the insurer’s role, confirming the connection between insurer actions and successful occupational rehabilitation and by revealing which actions best promote success.

Our results illustrate the multidimensional nature of the insurer’s role. Exploratory factor analysis suggested four dimensions for insurer actions. This was tested using confirmatory factor analysis, and factor scores from our final confirmatory model were used as input in a binary regression model. The first objective of the study was to examine the possible connection between insurer actions and the outcome of occupational rehabilitation. According to the research hypotheses, this type of connection would exist (H_1_), the claimant’s perceptions of positive insurer actions would be positively related to the success (H_2_) and the positive relationship would remain even if an adjustment was made for all likely confounding variables (H_3_). All these hypotheses were supported. The second objective of the study was to explore which insurer actions best promote success.

To test these hypotheses, we first used factor analysis to determine the dimensions of the insurer’s role. We came to four dimensions: (1) informing and guidance, (2) customer orientation, (3) process flow and (4) service attitude. Second, we used binary regression to explain the result of rehabilitation by factor scores. The results showed that sufficient, adequate, accessible, understandable, and professional informing and guidance is one significant factor promoting a positive outcome of rehabilitation. The importance of high-quality information and professional expertise has been brought up by various scholars [13, 32], and our results support and validate these previous findings.

The insurer’s customer-oriented approach, which includes paying attention to customer needs and involving customers in the planning and decision-making processes, had a significant positive effect on the outcome of rehabilitation. While the importance of customer-oriented approach has been highlighted in previous studies [7, 27, 32], our study suggests that changing the claimant’s role from a passive object to an active player is the most effective way for the insurer to promote success. Thus, customer-orientation should be highlighted in policy design, and new ways of making the claimants participate in the design and implementation of the rehabilitation process should be developed.

Several authors report friction between injured workers and insurers caused by delays in claims handling or claims manipulation [10, 33, 34], which can be expected to have a negative impact on the return-to-work process. Besides discrepancies in the compensation process, the wrong timing of rehabilitation has also proven to be harmful to a successful return to work [28, 35, 36] and vice versa. In accordance with these earlier results, the present study showed that a smooth process flow, including effective claims handling, justified decisions and timely actions, had a significant positive effect on the success of rehabilitation. This effect was considerably large, and alongside the customer-oriented approach, the smooth process flow seems to be one of the most important ways for insurers to contribute to successful rehabilitation. Thus, insurers would benefit from developing the compensation and rehabilitation processes as efficient and consistent as possible. Designated case managers within an insurance company could also improve the process flow.

Earlier studies have reported both negative [32, 33] and positive [14, 16, 37] experiences related to the insurer’s customer service. Olsson et al. [21] showed that supportive, encouraging and kind service was significantly associated with promoting the ability to RTW. We found that the service attitude factor had a small, but positive and statistically significant effect on the success of rehabilitation. However, it is noteworthy that the original variable relating to the kindness of service was highly skewed (− 1.163), indicating that the majority of the respondents received friendly service.

We also tested whether confounding variables could explain the connection between insurer actions and the rehabilitation outcome. The results showed that besides insurer actions, also age, self-rated ability to work before rehabilitation, motivation and rehabilitation objectives were statistically significant predictors of the rehabilitation outcome. When adjusting for potential confounding variables, all insurer factors remained statistically significant and the changes in the regression coefficients were minor.

### Research limitations and reliability

The questionnaire was created purely for this study. It was built and the hypotheses were formulated on the basis of previous literature. However, there were limitations concerning the development and testing of models. First, the questionnaire was part of a larger survey related to the functioning of occupational rehabilitation. Thus, the focus of the researchers or respondents was not limited to this instrument. Second, the questionnaire had not been used or tested before. However, it was reviewed by external researchers and the steering group of the rehabilitation service network. After receiving feedback, the comments were evaluated, and the questionnaire was revised.

The empirical testing was based on cross-sectional data gathered after occupational rehabilitation. Thus, memory errors and opinion changes are possible, which should be considered when interpreting the results. Furthermore, the Likert scale-based answer options of the survey questions might have been interpreted differently by the respondents. This tendency of giving systematically better or worse assessments on every question was taken into account in our final model by adding an individual effect factor (G). All criteria set for the validity of the factor model were met and all the hypotheses were supported by the data. In addition, the developed model helped verify the results of the earlier studies.

The other factors undermining the reliability of the research are related to the representativeness and suitability of the research data and the reliability of the used methods. The final data set included 661 respondents and the response rate was 29%, which is quite typical of an online survey. In surveys, non-response always occurs, and it is difficult to set unambiguous criteria for acceptable loss. Yet, non-response bias and representation of the population of interest should be evaluated. Table 1 shows the differences between the whole sample and the respondents. Although the proportion of respondents was higher in women than in men and higher in younger people than in elderly, the differences in gender and age between the respondents and those in the intended sample were minor. Thus, in the light of these variables, we consider that the respondents represent the whole sample at a satisfactory level.

Regarding the representation of the population of interest, we compared our actual sample to the population of all rehabilitees within an earnings-related pension in Finland in 2015 [38]. The distributions of the basic characteristics (age, gender, cause of disability) were quite similar, the main differences being that men and those suffering from musculoskeletal disorders were slightly over-represented in our sample, and those suffering from mental disorders were slightly under-represented.

However, the respondents could vary from these populations on an innumerable number of other factors that have not been tested. One major consideration is that our study sample only included rehabilitees who had a contact with a rehabilitation service provider. This choice is not random: a service provider is not usually involved in cases which are straightforward or easy to carry through. Therefore, our results may not fully generalize to a wider population of disabled workers.

Regarding the actual sample size, it can be considered sufficient for the methods used. Musil et al. [39] suggested that a structural equation model (SEM) analysis should have at least five observations for each variable. This condition was well fulfilled. However, it is a limitation that EFA and CFA were performed using the same data. Ideally, the factor model suggested by EFA would be tested with a new data set. In any case, there is a need to continue developing the model and to test its validity with different data.

Finally, the path coefficients in our two-stage model (in Fig. 2) should be interpreted with caution. Although the model is presented as a path diagram, and it is highly probable that there are causal links between insurer actions and the outcome of the rehabilitation, we do not consider the coefficients to be unbiased estimates of the actual causal effects. The reason is that the rehabilitee evaluated the performance of the insurer and the outcome of the rehabilitation at the same time, so that that feedback effects are likely to bias the estimates. Rather, the coefficients indicate the strength of association, or correlation, between the factors and the outcome of rehabilitation.

## Conclusions

Our results suggest that the insurer’s actions are substantially associated with the outcome of occupational rehabilitation. These findings support and confirm previous research results and help assess the relative importance of different actions by providing effect estimates for them. As for practical implications, the study reveals that the insurer would benefit most from ensuring a smooth process flow and adopting a customer-oriented approach, since they are the most important insurer-related determinants of successful occupational rehabilitation.

## Supplementary Information

**Additional file 1.**

## Data Availability

The datasets used and/or analysed during the current study are available from the corresponding author on reasonable request.

## Footnotes

1 Private sector wage earners are insured according to the Employees Pensions Act (TyEL) and constitute almost 70% of all wage earners in Finland [24].

2 Historically, the Finnish school grading system had a scale of 0 to 10, but all grades lower than 4 were consolidated into one failing grade of 4 in 1952.
