# Supplementary material for: How can social insurers promote return to work in occupational rehabilitation? A quantitative, cross-sectional study
Source: BMC Public Health. 2021 Sep 16;21:1687. doi: 10.1186/s12889-021-11758-w (PMC8444422; doi:10.1186/s12889-021-11758-w)
Supplement: Supplementary file 1 — Additional file 1. [file 12889_2021_11758_MOESM1_ESM.docx]

Success of rehabilitation

Arto Luoma [arto.luoma@wippies.com](mailto:arto.luoma@wippies.com), Jarna Pasanen [jarna.pasanen@tuni.fi](mailto:jarna.pasanen@tuni.fi)

30/6/2021

Statistical analysis for the article:

How can social insurers promote return to work in occupational rehabilitation? A quantitative, cross-sectional study. *BMC Public Health*.

Loading the needed libraries

**library**(lavaan)

**library**(semPlot)

**library**(tidyverse)

**library**(magrittr)

**library**(rcompanion)

Reading and manipulating the data

setwd("~/tyo/tutkimus/jarna/hanke2") *#setting the working library*

*#data <- read.csv2("Rehabilitation_surveydata2017.csv") #reading the data (all variables)*

data <- read.csv2("Data040621.csv") *#reading the data (a subset)*

*#“doesn’t concern me” is recoded to a missing value.*

data %<>%

mutate_at(c("Q19.1","Q19.2","Q19.3","Q19.4","Q19.5","Q19.6","Q19.7","Q19.8","Q19.10","Q19.11","Q23.1","Q23.2","Q23.3","Q23.4","Q23.5","Q23.6","Q23.12"),**function**(x){ifelse(x==6,NA,x)})

data %<>%

mutate(Q7=ifelse(Q7==7,NA,Q7))

*#Some of the variables are converted to numerical and some categorical*

data %<>% mutate(Q1 = as.numeric(Q1)-1,

Q9 = as.factor(Q9),

Q12.1 = as.numeric(Q12.1))

*#Creating the variables that describe the success of rehabilitation, age variable, average response level and its standardized version*

data %<>% mutate(RTW = ifelse(Q30 %**in**% 2:4,1,0), *#RTW: return to work*

SRH = ifelse(Q29>=4,1,0),*#SRH: subjective result of rehabilitation*

SUCC = ifelse(RTW==0 & SRH==0,0,1), *#SUCC: success of rehabilitation*

age = 2017-Q2,

avg = (Q19.1+ Q19.2 +Q19.3+ Q19.4+Q19.5+ Q19.6+ Q19.7+ Q19.8+

Q19.10+Q19.11+Q23.1+Q23.2+Q23.3+Q23.4+

Q23.5+Q23.6+Q23.12)/17,

avg.std = scale(avg) )

Exploratory factor analysis and regression analysis

*#Factor analysis with the maximum likelihood method and promax rotation*

fit_factanal <- factanal(~ Q19.1+ Q19.2 +Q19.3+ Q19.4+Q19.5+ Q19.6+ Q19.7+

Q19.8+Q19.10+Q19.11+Q23.1+Q23.2+Q23.3+Q23.4+

Q23.5+Q23.6+Q23.12,

factors=4,data=data,scores="Bartlett",na.action=na.exclude,rotation="promax")

print(fit_factanal)

##

## Call:

## factanal(x = ~Q19.1 + Q19.2 + Q19.3 + Q19.4 + Q19.5 + Q19.6 + Q19.7 + Q19.8 + Q19.10 + Q19.11 + Q23.1 + Q23.2 + Q23.3 + Q23.4 + Q23.5 + Q23.6 + Q23.12, factors = 4, data = data, na.action = na.exclude, scores = "Bartlett", rotation = "promax")

##

## Uniquenesses:

## Q19.1 Q19.2 Q19.3 Q19.4 Q19.5 Q19.6 Q19.7 Q19.8 Q19.10 Q19.11 Q23.1

## 0.105 0.088 0.075 0.068 0.087 0.085 0.101 0.005 0.079 0.156 0.117

## Q23.2 Q23.3 Q23.4 Q23.5 Q23.6 Q23.12

## 0.064 0.050 0.060 0.134 0.093 0.097

##

## Loadings:

## Factor1 Factor2 Factor3 Factor4

## Q19.1 0.894

## Q19.2 0.934

## Q19.3 0.928

## Q19.4 0.915

## Q19.5 0.976

## Q19.6 0.913

## Q19.7 0.878

## Q19.8 0.979

## Q19.10 0.893

## Q19.11 0.839

## Q23.1 0.917

## Q23.2 0.927

## Q23.3 0.971

## Q23.4 0.969

## Q23.5 0.835

## Q23.6 0.919

## Q23.12 0.931

##

## Factor1 Factor2 Factor3 Factor4

## SS loadings 5.097 4.424 3.240 1.670

## Proportion Var 0.300 0.260 0.191 0.098

## Cumulative Var 0.300 0.560 0.751 0.849

##

## Factor Correlations:

## Factor1 Factor2 Factor3 Factor4

## Factor1 1.000 0.540 0.692 -0.513

## Factor2 0.540 1.000 0.671 -0.707

## Factor3 0.692 0.671 1.000 -0.713

## Factor4 -0.513 -0.707 -0.713 1.000

##

## Test of the hypothesis that 4 factors are sufficient.

## The chi square statistic is 207.44 on 74 degrees of freedom.

## The p-value is 1.37e-14

*#Logistic regression model in which the success is explained by factor scores*

fit_glm <- glm(SUCC~fit_factanal$scores,data=data,family=binomial)

summary(fit_glm)

##

## Call:

## glm(formula = SUCC ~ fit_factanal$scores, family = binomial,

## data = data)

##

## Deviance Residuals:

## Min 1Q Median 3Q Max

## -3.2043 -0.2825 0.2190 0.4705 2.4216

##

## Coefficients:

## Estimate Std. Error z value Pr(>|z|)

## (Intercept) 0.2846 0.1404 2.027 0.042710 *

## fit_factanal$scoresFactor1 -0.9707 0.2517 -3.857 0.000115 ***

## fit_factanal$scoresFactor2 2.8716 0.2814 10.205 < 2e-16 ***

## fit_factanal$scoresFactor3 1.6957 0.2388 7.102 1.23e-12 ***

## fit_factanal$scoresFactor4 -1.5054 0.2557 -5.888 3.91e-09 ***

## ---

## Signif. codes: 0 '***' 0.001 '**' 0.01 '*' 0.05 '.' 0.1 ' ' 1

##

## (Dispersion parameter for binomial family taken to be 1)

##

## Null deviance: 778.22 on 573 degrees of freedom

## Residual deviance: 356.63 on 569 degrees of freedom

## (87 observations deleted due to missingness)

## AIC: 366.63

##

## Number of Fisher Scoring iterations: 6

The results are surprising and counter-intuitive, since factors 1 and 4 obtain negative coefficients. Perhaps there are confounding variables that have a causal effect on both the outcome and these factors. We try to add predictors to the data: age, ability to work before rehabilitation, motivation, the objectives of rehabilitation to improve professional skills or to receive a disability pension.

fit_glm2 <- glm(SUCC~fit_factanal$scores+age+Q10+Q11+Q12.4+Q12.6,data=data, family=binomial)

summary(fit_glm2)

##

## Call:

## glm(formula = SUCC ~ fit_factanal$scores + age + Q10 + Q11 +

## Q12.4 + Q12.6, family = binomial, data = data)

##

## Deviance Residuals:

## Min 1Q Median 3Q Max

## -3.2091 -0.2192 0.1769 0.4220 3.0145

##

## Coefficients:

## Estimate Std. Error z value Pr(>|z|)

## (Intercept) 0.05172 1.49078 0.035 0.972325

## fit_factanal$scoresFactor1 -0.92135 0.26587 -3.465 0.000529 ***

## fit_factanal$scoresFactor2 2.56504 0.29366 8.735 < 2e-16 ***

## fit_factanal$scoresFactor3 1.72403 0.25359 6.798 1.06e-11 ***

## fit_factanal$scoresFactor4 -1.44457 0.27421 -5.268 1.38e-07 ***

## age -0.04446 0.01952 -2.277 0.022757 *

## Q10 0.22946 0.10931 2.099 0.035801 *

## Q11 0.26392 0.19418 1.359 0.174091

## Q12.4 0.36295 0.14514 2.501 0.012395 *

## Q12.6 -0.27442 0.10174 -2.697 0.006991 **

## ---

## Signif. codes: 0 '***' 0.001 '**' 0.01 '*' 0.05 '.' 0.1 ' ' 1

##

## (Dispersion parameter for binomial family taken to be 1)

##

## Null deviance: 759.09 on 559 degrees of freedom

## Residual deviance: 311.61 on 550 degrees of freedom

## (101 observations deleted due to missingness)

## AIC: 331.61

##

## Number of Fisher Scoring iterations: 6

The regression coefficients of the factors hardly change

We add an “average effect” or “individual effect” to the model

fit_glm3 <- glm(SUCC~fit_factanal$scores[,1:3]+avg.std+age+Q10+Q12.4+Q12.6,data=data, family=binomial)

summary(fit_glm3)

##

## Call:

## glm(formula = SUCC ~ fit_factanal$scores[, 1:3] + avg.std + age +

## Q10 + Q12.4 + Q12.6, family = binomial, data = data)

##

## Deviance Residuals:

## Min 1Q Median 3Q Max

## -3.2805 -0.2209 0.1840 0.4321 3.0094

##

## Coefficients:

## Estimate Std. Error z value Pr(>|z|)

## (Intercept) 0.88204 1.27590 0.691 0.489373

## fit_factanal$scores[, 1:3]Factor1 3.67607 0.96762 3.799 0.000145 ***

## fit_factanal$scores[, 1:3]Factor2 6.55127 0.91654 7.148 8.82e-13 ***

## fit_factanal$scores[, 1:3]Factor3 4.61778 0.65720 7.026 2.12e-12 ***

## avg.std -11.29962 2.10578 -5.366 8.05e-08 ***

## age -0.04128 0.01955 -2.111 0.034756 *

## Q10 0.23742 0.10967 2.165 0.030394 *

## Q12.4 0.42202 0.13872 3.042 0.002349 **

## Q12.6 -0.31131 0.10096 -3.084 0.002046 **

## ---

## Signif. codes: 0 '***' 0.001 '**' 0.01 '*' 0.05 '.' 0.1 ' ' 1

##

## (Dispersion parameter for binomial family taken to be 1)

##

## Null deviance: 761.92 on 561 degrees of freedom

## Residual deviance: 312.14 on 553 degrees of freedom

## (99 observations deleted due to missingness)

## AIC: 330.14

##

## Number of Fisher Scoring iterations: 6

We see that when the average effect is taken out, factors 1 to 3 have positive coefficients. If all factors 1 to 4 were included in the model the predictors would be nearly collinear.

We will proceed with confirmatory factor analysis and structural equation modelling

SEM analysis with lavaan

Confirmatory factor analysis (CFA)

*#Model with a free correlation structure of factors*

cfa_model1 <- 'F1 =~ Q23.6 + Q19.7 + Q19.4 + Q19.5 + Q19.6 + Q23.1

F2 =~ Q23.4 + Q23.3 + Q23.2 + Q23.12 + Q19.10

F3 =~ Q19.3 + Q19.2 + Q19.1 + Q23.5

F4 =~ Q19.8 + Q19.11'

fit_cfa_model1 <- lavaan(cfa_model1,data=data,auto.var=TRUE,auto.cov.lv.x = TRUE,

std.ov=TRUE,std.lv=TRUE,missing="ml")

## Warning in lav_data_full(data = data, group = group, cluster = cluster, : lavaan WARNING: some cases are empty and will be ignored:

## 414

summary(fit_cfa_model1, fit.measures = TRUE)

## lavaan 0.6-7 ended normally after 78 iterations

##

## Estimator ML

## Optimization method NLMINB

## Number of free parameters 40

##

## Used Total

## Number of observations 660 661

## Number of missing patterns 54

##

## Model Test User Model:

##

## Test statistic 308.970

## Degrees of freedom 130

## P-value (Chi-square) 0.000

##

## Model Test Baseline Model:

##

## Test statistic 18547.471

## Degrees of freedom 136

## P-value 0.000

##

## User Model versus Baseline Model:

##

## Comparative Fit Index (CFI) 0.990

## Tucker-Lewis Index (TLI) 0.990

##

## Loglikelihood and Information Criteria:

##

## Loglikelihood user model (H0) -6334.416

## Loglikelihood unrestricted model (H1) -6179.931

##

## Akaike (AIC) 12748.832

## Bayesian (BIC) 12928.522

## Sample-size adjusted Bayesian (BIC) 12801.521

##

## Root Mean Square Error of Approximation:

##

## RMSEA 0.046

## 90 Percent confidence interval - lower 0.039

## 90 Percent confidence interval - upper 0.052

## P-value RMSEA <= 0.05 0.857

##

## Standardized Root Mean Square Residual:

##

## SRMR 0.014

##

## Parameter Estimates:

##

## Standard errors Standard

## Information Observed

## Observed information based on Hessian

##

## Latent Variables:

## Estimate Std.Err z-value P(>|z|)

## F1 =~

## Q23.6 0.942 0.029 32.643 0.000

## Q19.7 0.943 0.029 32.626 0.000

## Q19.4 0.957 0.028 33.734 0.000

## Q19.5 0.949 0.029 32.883 0.000

## Q19.6 0.949 0.029 33.146 0.000

## Q23.1 0.936 0.029 31.874 0.000

## F2 =~

## Q23.4 0.968 0.029 33.693 0.000

## Q23.3 0.970 0.029 33.999 0.000

## Q23.2 0.963 0.029 33.471 0.000

## Q23.12 0.950 0.029 32.697 0.000

## Q19.10 0.949 0.029 33.046 0.000

## F3 =~

## Q19.3 0.957 0.029 33.356 0.000

## Q19.2 0.953 0.029 32.830 0.000

## Q19.1 0.941 0.029 32.412 0.000

## Q23.5 0.930 0.030 31.377 0.000

## F4 =~

## Q19.8 0.964 0.030 32.524 0.000

## Q19.11 0.933 0.030 31.246 0.000

##

## Covariances:

## Estimate Std.Err z-value P(>|z|)

## F1 ~~

## F2 0.681 0.022 31.367 0.000

## F3 0.746 0.018 40.808 0.000

## F4 0.741 0.019 38.945 0.000

## F2 ~~

## F3 0.741 0.019 39.841 0.000

## F4 0.549 0.029 19.065 0.000

## F3 ~~

## F4 0.561 0.028 19.774 0.000

##

## Intercepts:

## Estimate Std.Err z-value P(>|z|)

## .Q23.6 0.000

## .Q19.7 0.000

## .Q19.4 0.000

## .Q19.5 0.000

## .Q19.6 0.000

## .Q23.1 0.000

## .Q23.4 0.000

## .Q23.3 0.000

## .Q23.2 0.000

## .Q23.12 0.000

## .Q19.10 0.000

## .Q19.3 0.000

## .Q19.2 0.000

## .Q19.1 0.000

## .Q23.5 0.000

## .Q19.8 0.000

## .Q19.11 0.000

## F1 0.000

## F2 0.000

## F3 0.000

## F4 0.000

##

## Variances:

## Estimate Std.Err z-value P(>|z|)

## .Q23.6 0.101 0.007 15.134 0.000

## .Q19.7 0.103 0.007 15.330 0.000

## .Q19.4 0.070 0.005 13.836 0.000

## .Q19.5 0.095 0.006 15.071 0.000

## .Q19.6 0.086 0.006 14.728 0.000

## .Q23.1 0.124 0.008 15.657 0.000

## .Q23.4 0.066 0.005 13.696 0.000

## .Q23.3 0.055 0.004 12.736 0.000

## .Q23.2 0.073 0.005 14.095 0.000

## .Q23.12 0.096 0.006 15.128 0.000

## .Q19.10 0.082 0.006 14.449 0.000

## .Q19.3 0.079 0.006 12.239 0.000

## .Q19.2 0.094 0.007 13.193 0.000

## .Q19.1 0.106 0.008 13.804 0.000

## .Q23.5 0.136 0.009 14.789 0.000

## .Q19.8 0.067 0.016 4.179 0.000

## .Q19.11 0.107 0.016 6.775 0.000

## F1 1.000

## F2 1.000

## F3 1.000

## F4 1.000

*#Model with uncorrelated factors*

cfa_model2 <- 'F1 =~ Q23.6 + Q19.7 + Q19.4 + Q19.5 + Q19.6 + Q23.1

F2 =~ Q23.4 + Q23.3 + Q23.2 + Q23.12 + Q19.10

F3 =~ Q19.3 + Q19.2 + Q19.1 + Q23.5

F4 =~ b*Q19.8 + b*Q19.11'

fit_cfa_model2 <- lavaan(cfa_model2,data=data,auto.var=TRUE,auto.cov.lv.x = FALSE,

std.ov=TRUE,std.lv=TRUE,missing="ml")

## Warning in lav_data_full(data = data, group = group, cluster = cluster, : lavaan WARNING: some cases are empty and will be ignored:

## 414

summary(fit_cfa_model2, fit.measures = TRUE)

## lavaan 0.6-7 ended normally after 71 iterations

##

## Estimator ML

## Optimization method NLMINB

## Number of free parameters 34

## Number of equality constraints 1

##

## Used Total

## Number of observations 660 661

## Number of missing patterns 54

##

## Model Test User Model:

##

## Test statistic 1827.593

## Degrees of freedom 137

## P-value (Chi-square) 0.000

##

## Model Test Baseline Model:

##

## Test statistic 18547.471

## Degrees of freedom 136

## P-value 0.000

##

## User Model versus Baseline Model:

##

## Comparative Fit Index (CFI) 0.908

## Tucker-Lewis Index (TLI) 0.909

##

## Loglikelihood and Information Criteria:

##

## Loglikelihood user model (H0) -7093.728

## Loglikelihood unrestricted model (H1) -6179.931

##

## Akaike (AIC) 14253.455

## Bayesian (BIC) 14401.699

## Sample-size adjusted Bayesian (BIC) 14296.923

##

## Root Mean Square Error of Approximation:

##

## RMSEA 0.137

## 90 Percent confidence interval - lower 0.131

## 90 Percent confidence interval - upper 0.142

## P-value RMSEA <= 0.05 0.000

##

## Standardized Root Mean Square Residual:

##

## SRMR 0.496

##

## Parameter Estimates:

##

## Standard errors Standard

## Information Observed

## Observed information based on Hessian

##

## Latent Variables:

## Estimate Std.Err z-value P(>|z|)

## F1 =~

## Q23.6 0.943 0.029 32.587 0.000

## Q19.7 0.943 0.029 32.514 0.000

## Q19.4 0.958 0.028 33.634 0.000

## Q19.5 0.951 0.029 32.906 0.000

## Q19.6 0.950 0.029 33.066 0.000

## Q23.1 0.938 0.029 31.819 0.000

## F2 =~

## Q23.4 0.966 0.029 33.706 0.000

## Q23.3 0.969 0.028 34.002 0.000

## Q23.2 0.960 0.029 33.380 0.000

## Q23.12 0.949 0.029 32.678 0.000

## Q19.10 0.947 0.029 32.952 0.000

## F3 =~

## Q19.3 0.959 0.029 33.342 0.000

## Q19.2 0.956 0.029 32.793 0.000

## Q19.1 0.942 0.029 32.291 0.000

## Q23.5 0.929 0.030 31.143 0.000

## F4 =~

## Q19.8 (b) 0.951 0.028 34.277 0.000

## Q19.11 (b) 0.951 0.028 34.277 0.000

##

## Intercepts:

## Estimate Std.Err z-value P(>|z|)

## .Q23.6 0.000

## .Q19.7 0.000

## .Q19.4 0.000

## .Q19.5 0.000

## .Q19.6 0.000

## .Q23.1 0.000

## .Q23.4 0.000

## .Q23.3 0.000

## .Q23.2 0.000

## .Q23.12 0.000

## .Q19.10 0.000

## .Q19.3 0.000

## .Q19.2 0.000

## .Q19.1 0.000

## .Q23.5 0.000

## .Q19.8 0.000

## .Q19.11 0.000

## F1 0.000

## F2 0.000

## F3 0.000

## F4 0.000

##

## Variances:

## Estimate Std.Err z-value P(>|z|)

## .Q23.6 0.101 0.007 14.998 0.000

## .Q19.7 0.104 0.007 15.267 0.000

## .Q19.4 0.070 0.005 13.717 0.000

## .Q19.5 0.092 0.006 14.839 0.000

## .Q19.6 0.087 0.006 14.599 0.000

## .Q23.1 0.124 0.008 15.549 0.000

## .Q23.4 0.064 0.005 13.478 0.000

## .Q23.3 0.054 0.004 12.486 0.000

## .Q23.2 0.075 0.005 14.102 0.000

## .Q23.12 0.095 0.006 15.047 0.000

## .Q19.10 0.084 0.006 14.468 0.000

## .Q19.3 0.077 0.007 11.689 0.000

## .Q19.2 0.092 0.007 12.802 0.000

## .Q19.1 0.107 0.008 13.596 0.000

## .Q23.5 0.141 0.010 14.787 0.000

## .Q19.8 0.096 0.017 5.672 0.000

## .Q19.11 0.078 0.017 4.651 0.000

## F1 1.000

## F2 1.000

## F3 1.000

## F4 1.000

*#Model with an 'individual effect' G*

cfa_model3 <- 'F1 =~ Q23.6 + Q19.7 + Q19.4 + Q19.5 + Q19.6 + Q23.1

F2 =~ Q23.4 + Q23.3 + Q23.2 + Q23.12 + Q19.10

F3 =~ Q19.3 + Q19.2 + Q19.1 + Q23.5

F4 =~ b*Q19.8 + b*Q19.11

G =~ a*Q23.6 + a*Q19.7 + a*Q19.4 + a*Q19.5 + a*Q19.6 + a*Q23.1 +

a*Q23.4 + a*Q23.3 + a*Q23.2 + a*Q23.12 + a*Q19.10 +

a*Q19.3 + a*Q19.2 + a*Q19.1 + a*Q23.5 +

a*Q19.8 + a*Q19.11'

fit_cfa_model3 <- lavaan(cfa_model3,data=data,auto.var=TRUE,std.ov=TRUE,std.lv=TRUE,missing="ml")

## Warning in lav_data_full(data = data, group = group, cluster = cluster, : lavaan WARNING: some cases are empty and will be ignored:

## 414

summary(fit_cfa_model3,fit.measures=TRUE)

## lavaan 0.6-7 ended normally after 77 iterations

##

## Estimator ML

## Optimization method NLMINB

## Number of free parameters 51

## Number of equality constraints 17

##

## Used Total

## Number of observations 660 661

## Number of missing patterns 54

##

## Model Test User Model:

##

## Test statistic 388.444

## Degrees of freedom 136

## P-value (Chi-square) 0.000

##

## Model Test Baseline Model:

##

## Test statistic 18547.471

## Degrees of freedom 136

## P-value 0.000

##

## User Model versus Baseline Model:

##

## Comparative Fit Index (CFI) 0.986

## Tucker-Lewis Index (TLI) 0.986

##

## Loglikelihood and Information Criteria:

##

## Loglikelihood user model (H0) -6374.153

## Loglikelihood unrestricted model (H1) -6179.931

##

## Akaike (AIC) 12816.306

## Bayesian (BIC) 12969.042

## Sample-size adjusted Bayesian (BIC) 12861.091

##

## Root Mean Square Error of Approximation:

##

## RMSEA 0.053

## 90 Percent confidence interval - lower 0.047

## 90 Percent confidence interval - upper 0.059

## P-value RMSEA <= 0.05 0.204

##

## Standardized Root Mean Square Residual:

##

## SRMR 0.059

##

## Parameter Estimates:

##

## Standard errors Standard

## Information Observed

## Observed information based on Hessian

##

## Latent Variables:

## Estimate Std.Err z-value P(>|z|)

## F1 =~

## Q23.6 0.429 0.025 17.151 0.000

## Q19.7 0.422 0.025 16.860 0.000

## Q19.4 0.441 0.023 18.870 0.000

## Q19.5 0.470 0.025 19.082 0.000

## Q19.6 0.437 0.024 18.045 0.000

## Q23.1 0.426 0.026 16.209 0.000

## F2 =~

## Q23.4 0.598 0.024 24.695 0.000

## Q23.3 0.601 0.024 25.145 0.000

## Q23.2 0.559 0.024 23.377 0.000

## Q23.12 0.576 0.025 22.946 0.000

## Q19.10 0.534 0.024 22.330 0.000

## F3 =~

## Q19.3 0.534 0.024 22.083 0.000

## Q19.2 0.521 0.025 21.255 0.000

## Q19.1 0.501 0.025 20.054 0.000

## Q23.5 0.467 0.026 17.960 0.000

## F4 =~

## Q19.8 (b) 0.623 0.024 26.385 0.000

## Q19.11 (b) 0.623 0.024 26.385 0.000

## G =~

## Q23.6 (a) 0.797 0.025 32.315 0.000

## Q19.7 (a) 0.797 0.025 32.315 0.000

## Q19.4 (a) 0.797 0.025 32.315 0.000

## Q19.5 (a) 0.797 0.025 32.315 0.000

## Q19.6 (a) 0.797 0.025 32.315 0.000

## Q23.1 (a) 0.797 0.025 32.315 0.000

## Q23.4 (a) 0.797 0.025 32.315 0.000

## Q23.3 (a) 0.797 0.025 32.315 0.000

## Q23.2 (a) 0.797 0.025 32.315 0.000

## Q23.12 (a) 0.797 0.025 32.315 0.000

## Q19.10 (a) 0.797 0.025 32.315 0.000

## Q19.3 (a) 0.797 0.025 32.315 0.000

## Q19.2 (a) 0.797 0.025 32.315 0.000

## Q19.1 (a) 0.797 0.025 32.315 0.000

## Q23.5 (a) 0.797 0.025 32.315 0.000

## Q19.8 (a) 0.797 0.025 32.315 0.000

## Q19.11 (a) 0.797 0.025 32.315 0.000

##

## Intercepts:

## Estimate Std.Err z-value P(>|z|)

## .Q23.6 0.000

## .Q19.7 0.000

## .Q19.4 0.000

## .Q19.5 0.000

## .Q19.6 0.000

## .Q23.1 0.000

## .Q23.4 0.000

## .Q23.3 0.000

## .Q23.2 0.000

## .Q23.12 0.000

## .Q19.10 0.000

## .Q19.3 0.000

## .Q19.2 0.000

## .Q19.1 0.000

## .Q23.5 0.000

## .Q19.8 0.000

## .Q19.11 0.000

## F1 0.000

## F2 0.000

## F3 0.000

## F4 0.000

## G 0.000

##

## Variances:

## Estimate Std.Err z-value P(>|z|)

## .Q23.6 0.101 0.007 15.081 0.000

## .Q19.7 0.104 0.007 15.423 0.000

## .Q19.4 0.070 0.005 13.766 0.000

## .Q19.5 0.091 0.006 14.257 0.000

## .Q19.6 0.087 0.006 14.640 0.000

## .Q23.1 0.124 0.008 15.619 0.000

## .Q23.4 0.064 0.005 13.152 0.000

## .Q23.3 0.053 0.004 12.053 0.000

## .Q23.2 0.074 0.005 14.308 0.000

## .Q23.12 0.095 0.006 15.017 0.000

## .Q19.10 0.081 0.006 14.689 0.000

## .Q19.3 0.076 0.007 11.166 0.000

## .Q19.2 0.092 0.007 12.666 0.000

## .Q19.1 0.108 0.008 13.739 0.000

## .Q23.5 0.139 0.009 15.098 0.000

## .Q19.8 0.084 0.013 6.573 0.000

## .Q19.11 0.090 0.013 6.971 0.000

## F1 1.000

## F2 1.000

## F3 1.000

## F4 1.000

## G 1.000

Next, we add the outcome variable to the model

str_model <- 'SUCC ~ F1 + F2 + F3 + F4

F1 =~ Q23.6 + Q19.7 + Q19.4 + Q19.5 + Q19.6 + Q23.1

F2 =~ Q23.4 + Q23.3 + Q23.2 + Q23.12 + Q19.10

F3 =~ Q19.3 + Q19.2 + Q19.1 + Q23.5

F4 =~ Q19.8 + Q19.11

G =~ a*Q23.6 + a*Q19.7 + a*Q19.4 + a*Q19.5 + a*Q19.6 + a*Q23.1 +

a*Q23.4 + a*Q23.3 + a*Q23.2 + a*Q23.12 + a*Q19.10 +

a*Q19.3 + a*Q19.2 + a*Q19.1 + a*Q23.5 +

a*Q19.8 + a*Q19.11'

fit_str_model <- lavaan(str_model,data=data,ordered='SUCC',auto.var=TRUE,std.ov=TRUE,std.lv=TRUE)

## Warning in lav_object_post_check(object): lavaan WARNING: some estimated ov

## variances are negative

summary(fit_str_model,fit.measures=TRUE)

## lavaan 0.6-7 ended normally after 45 iterations

##

## Estimator DWLS

## Optimization method NLMINB

## Number of free parameters 55

## Number of equality constraints 16

##

## Used Total

## Number of observations 574 661

##

## Model Test User Model:

## Standard Robust

## Test Statistic 86.000 167.230

## Degrees of freedom 149 149

## P-value (Chi-square) 1.000 0.146

## Scaling correction factor 1.447

## Shift parameter 107.778

## simple second-order correction

##

## Model Test Baseline Model:

##

## Test statistic 13608.383 2028.158

## Degrees of freedom 153 153

## P-value 0.000 0.000

## Scaling correction factor 7.176

##

## User Model versus Baseline Model:

##

## Comparative Fit Index (CFI) 1.000 0.990

## Tucker-Lewis Index (TLI) 1.005 0.990

##

## Robust Comparative Fit Index (CFI) NA

## Robust Tucker-Lewis Index (TLI) NA

##

## Root Mean Square Error of Approximation:

##

## RMSEA 0.000 0.015

## 90 Percent confidence interval - lower 0.000 0.000

## 90 Percent confidence interval - upper 0.000 0.025

## P-value RMSEA <= 0.05 1.000 1.000

##

## Robust RMSEA NA

## 90 Percent confidence interval - lower 0.000

## 90 Percent confidence interval - upper NA

##

## Standardized Root Mean Square Residual:

##

## SRMR 0.046 0.046

##

## Parameter Estimates:

##

## Standard errors Robust.sem

## Information Expected

## Information saturated (h1) model Unstructured

##

## Latent Variables:

## Estimate Std.Err z-value P(>|z|)

## F1 =~

## Q23.6 0.533 0.062 8.624 0.000

## Q19.7 0.504 0.062 8.168 0.000

## Q19.4 0.540 0.064 8.438 0.000

## Q19.5 0.545 0.065 8.426 0.000

## Q19.6 0.520 0.062 8.394 0.000

## Q23.1 0.502 0.064 7.909 0.000

## F2 =~

## Q23.4 0.540 0.060 9.074 0.000

## Q23.3 0.553 0.061 9.026 0.000

## Q23.2 0.548 0.056 9.717 0.000

## Q23.12 0.531 0.059 8.952 0.000

## Q19.10 0.544 0.058 9.298 0.000

## F3 =~

## Q19.3 0.528 0.067 7.897 0.000

## Q19.2 0.516 0.060 8.589 0.000

## Q19.1 0.505 0.063 8.030 0.000

## Q23.5 0.505 0.057 8.879 0.000

## F4 =~

## Q19.8 0.504 0.066 7.692 0.000

## Q19.11 0.554 0.073 7.569 0.000

## G =~

## Q23.6 (a) 0.796 0.034 23.088 0.000

## Q19.7 (a) 0.796 0.034 23.088 0.000

## Q19.4 (a) 0.796 0.034 23.088 0.000

## Q19.5 (a) 0.796 0.034 23.088 0.000

## Q19.6 (a) 0.796 0.034 23.088 0.000

## Q23.1 (a) 0.796 0.034 23.088 0.000

## Q23.4 (a) 0.796 0.034 23.088 0.000

## Q23.3 (a) 0.796 0.034 23.088 0.000

## Q23.2 (a) 0.796 0.034 23.088 0.000

## Q23.12 (a) 0.796 0.034 23.088 0.000

## Q19.10 (a) 0.796 0.034 23.088 0.000

## Q19.3 (a) 0.796 0.034 23.088 0.000

## Q19.2 (a) 0.796 0.034 23.088 0.000

## Q19.1 (a) 0.796 0.034 23.088 0.000

## Q23.5 (a) 0.796 0.034 23.088 0.000

## Q19.8 (a) 0.796 0.034 23.088 0.000

## Q19.11 (a) 0.796 0.034 23.088 0.000

##

## Regressions:

## Estimate Std.Err z-value P(>|z|)

## SUCC ~

## F1 0.720 0.110 6.548 0.000

## F2 1.317 0.094 14.086 0.000

## F3 1.216 0.114 10.623 0.000

## F4 0.368 0.110 3.334 0.001

##

## Intercepts:

## Estimate Std.Err z-value P(>|z|)

## .Q23.6 0.000

## .Q19.7 0.000

## .Q19.4 0.000

## .Q19.5 0.000

## .Q19.6 0.000

## .Q23.1 0.000

## .Q23.4 0.000

## .Q23.3 0.000

## .Q23.2 0.000

## .Q23.12 0.000

## .Q19.10 0.000

## .Q19.3 0.000

## .Q19.2 0.000

## .Q19.1 0.000

## .Q23.5 0.000

## .Q19.8 0.000

## .Q19.11 0.000

## .SUCC 0.000

## F1 0.000

## F2 0.000

## F3 0.000

## F4 0.000

## G 0.000

##

## Thresholds:

## Estimate Std.Err z-value P(>|z|)

## SUCC|t1 -0.220

##

## Variances:

## Estimate Std.Err z-value P(>|z|)

## .Q23.6 0.081 0.018 4.553 0.000

## .Q19.7 0.111 0.024 4.732 0.000

## .Q19.4 0.074 0.017 4.249 0.000

## .Q19.5 0.068 0.019 3.676 0.000

## .Q19.6 0.095 0.020 4.791 0.000

## .Q23.1 0.113 0.024 4.757 0.000

## .Q23.4 0.074 0.022 3.348 0.001

## .Q23.3 0.059 0.021 2.804 0.005

## .Q23.2 0.065 0.021 3.158 0.002

## .Q23.12 0.083 0.028 2.936 0.003

## .Q19.10 0.070 0.029 2.447 0.014

## .Q19.3 0.087 0.024 3.614 0.000

## .Q19.2 0.099 0.024 4.171 0.000

## .Q19.1 0.110 0.026 4.165 0.000

## .Q23.5 0.110 0.026 4.166 0.000

## .Q19.8 0.111 0.037 2.968 0.003

## .Q19.11 0.059 0.044 1.332 0.183

## .SUCC -2.868

## F1 1.000

## F2 1.000

## F3 1.000

## F4 1.000

## G 1.000

##

## Scales y*:

## Estimate Std.Err z-value P(>|z|)

## SUCC 1.000

semPaths(fit_str_model,"est",intercepts=FALSE,residuals=FALSE)


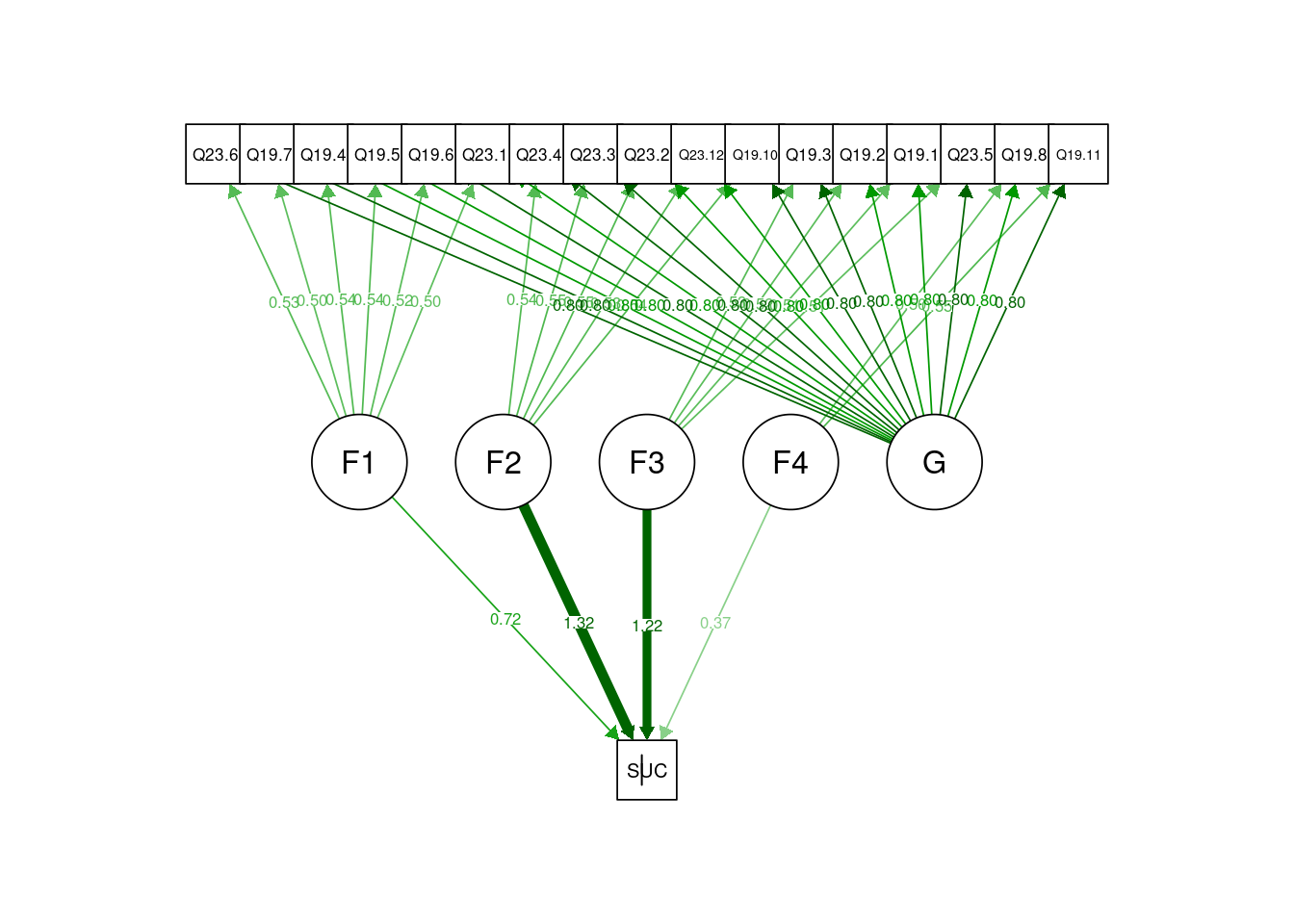


We fit a model with individual effect + factors + Q10, Q12.4, Q12.6

str_model2 <- 'SUCC ~ F1 + F2 + F3 + F4 + Q10 + Q12.4 + Q12.6

F1 =~ Q23.6 + Q19.7 + Q19.4 + Q19.5 + Q19.6 + Q23.1

F2 =~ Q23.4 + Q23.3 + Q23.2 + Q23.12 + Q19.10

F3 =~ Q19.3 + Q19.2 + Q19.1 + Q19.3 + Q23.5

F4 =~ Q19.8 + Q19.11

G =~ a*Q23.6 + a*Q19.7 + a*Q19.4 + a*Q19.5 + a*Q19.6 + a*Q23.1 +

a*Q23.4 + a*Q23.3 + a*Q23.2 + a*Q23.12 + a*Q19.10 +

a*Q19.3 + a*Q19.2 + a*Q19.1 + a*Q19.3 + a*Q23.5 +

a*Q19.8 + a*Q19.11'

fit_str_model2 <- lavaan(str_model2,data=data,ordered='SUCC',auto.var=TRUE,std.ov=TRUE,std.lv=TRUE)

## Warning in lav_object_post_check(object): lavaan WARNING: some estimated ov

## variances are negative

summary(fit_str_model2)

## lavaan 0.6-7 ended normally after 41 iterations

##

## Estimator DWLS

## Optimization method NLMINB

## Number of free parameters 58

## Number of equality constraints 16

##

## Used Total

## Number of observations 562 661

##

## Model Test User Model:

## Standard Robust

## Test Statistic 903.495 601.804

## Degrees of freedom 200 200

## P-value (Chi-square) 0.000 0.000

## Scaling correction factor 1.974

## Shift parameter 144.006

## simple second-order correction

##

## Parameter Estimates:

##

## Standard errors Robust.sem

## Information Expected

## Information saturated (h1) model Unstructured

##

## Latent Variables:

## Estimate Std.Err z-value P(>|z|)

## F1 =~

## Q23.6 0.541 0.057 9.431 0.000

## Q19.7 0.525 0.060 8.800 0.000

## Q19.4 0.564 0.059 9.640 0.000

## Q19.5 0.561 0.060 9.329 0.000

## Q19.6 0.546 0.058 9.340 0.000

## Q23.1 0.518 0.059 8.718 0.000

## F2 =~

## Q23.4 0.463 0.050 9.228 0.000

## Q23.3 0.464 0.051 9.194 0.000

## Q23.2 0.460 0.047 9.772 0.000

## Q23.12 0.454 0.050 9.096 0.000

## Q19.10 0.454 0.048 9.372 0.000

## F3 =~

## Q19.3 0.526 0.057 9.266 0.000

## Q19.2 0.505 0.051 9.930 0.000

## Q19.1 0.498 0.053 9.378 0.000

## Q23.5 0.497 0.049 10.120 0.000

## F4 =~

## Q19.8 0.558 0.073 7.652 0.000

## Q19.11 0.607 0.077 7.858 0.000

## G =~

## Q23.6 (a) 0.735 0.031 24.071 0.000

## Q19.7 (a) 0.735 0.031 24.071 0.000

## Q19.4 (a) 0.735 0.031 24.071 0.000

## Q19.5 (a) 0.735 0.031 24.071 0.000

## Q19.6 (a) 0.735 0.031 24.071 0.000

## Q23.1 (a) 0.735 0.031 24.071 0.000

## Q23.4 (a) 0.735 0.031 24.071 0.000

## Q23.3 (a) 0.735 0.031 24.071 0.000

## Q23.2 (a) 0.735 0.031 24.071 0.000

## Q23.12 (a) 0.735 0.031 24.071 0.000

## Q19.10 (a) 0.735 0.031 24.071 0.000

## Q19.3 (a) 0.735 0.031 24.071 0.000

## Q19.2 (a) 0.735 0.031 24.071 0.000

## Q19.1 (a) 0.735 0.031 24.071 0.000

## Q23.5 (a) 0.735 0.031 24.071 0.000

## Q19.8 (a) 0.735 0.031 24.071 0.000

## Q19.11 (a) 0.735 0.031 24.071 0.000

##

## Regressions:

## Estimate Std.Err z-value P(>|z|)

## SUCC ~

## F1 0.513 0.100 5.139 0.000

## F2 1.258 0.105 11.949 0.000

## F3 1.070 0.098 10.974 0.000

## F4 0.205 0.092 2.228 0.026

## Q10 0.311 0.053 5.823 0.000

## Q12.4 0.355 0.061 5.818 0.000

## Q12.6 -0.375 0.060 -6.203 0.000

##

## Intercepts:

## Estimate Std.Err z-value P(>|z|)

## .Q23.6 0.000

## .Q19.7 0.000

## .Q19.4 0.000

## .Q19.5 0.000

## .Q19.6 0.000

## .Q23.1 0.000

## .Q23.4 0.000

## .Q23.3 0.000

## .Q23.2 0.000

## .Q23.12 0.000

## .Q19.10 0.000

## .Q19.3 0.000

## .Q19.2 0.000

## .Q19.1 0.000

## .Q23.5 0.000

## .Q19.8 0.000

## .Q19.11 0.000

## .SUCC 0.000

## F1 0.000

## F2 0.000

## F3 0.000

## F4 0.000

## G 0.000

##

## Thresholds:

## Estimate Std.Err z-value P(>|z|)

## SUCC|t1 -0.252

##

## Variances:

## Estimate Std.Err z-value P(>|z|)

## .Q23.6 0.085 0.012 7.395 0.000

## .Q19.7 0.112 0.016 7.103 0.000

## .Q19.4 0.068 0.011 6.107 0.000

## .Q19.5 0.068 0.012 5.768 0.000

## .Q19.6 0.093 0.012 7.689 0.000

## .Q23.1 0.115 0.015 7.572 0.000

## .Q23.4 0.077 0.017 4.579 0.000

## .Q23.3 0.056 0.015 3.612 0.000

## .Q23.2 0.061 0.015 4.075 0.000

## .Q23.12 0.087 0.021 4.237 0.000

## .Q19.10 0.070 0.021 3.328 0.001

## .Q19.3 0.077 0.016 4.704 0.000

## .Q19.2 0.101 0.017 5.879 0.000

## .Q19.1 0.114 0.019 5.896 0.000

## .Q23.5 0.112 0.020 5.610 0.000

## .Q19.8 0.118 0.060 1.984 0.047

## .Q19.11 0.052 0.070 0.750 0.453

## .SUCC -2.033

## F1 1.000

## F2 1.000

## F3 1.000

## F4 1.000

## G 1.000

##

## Scales y*:

## Estimate Std.Err z-value P(>|z|)

## SUCC 1.000

semPaths(fit_str_model2,"est",intercepts=FALSE,residuals=FALSE)


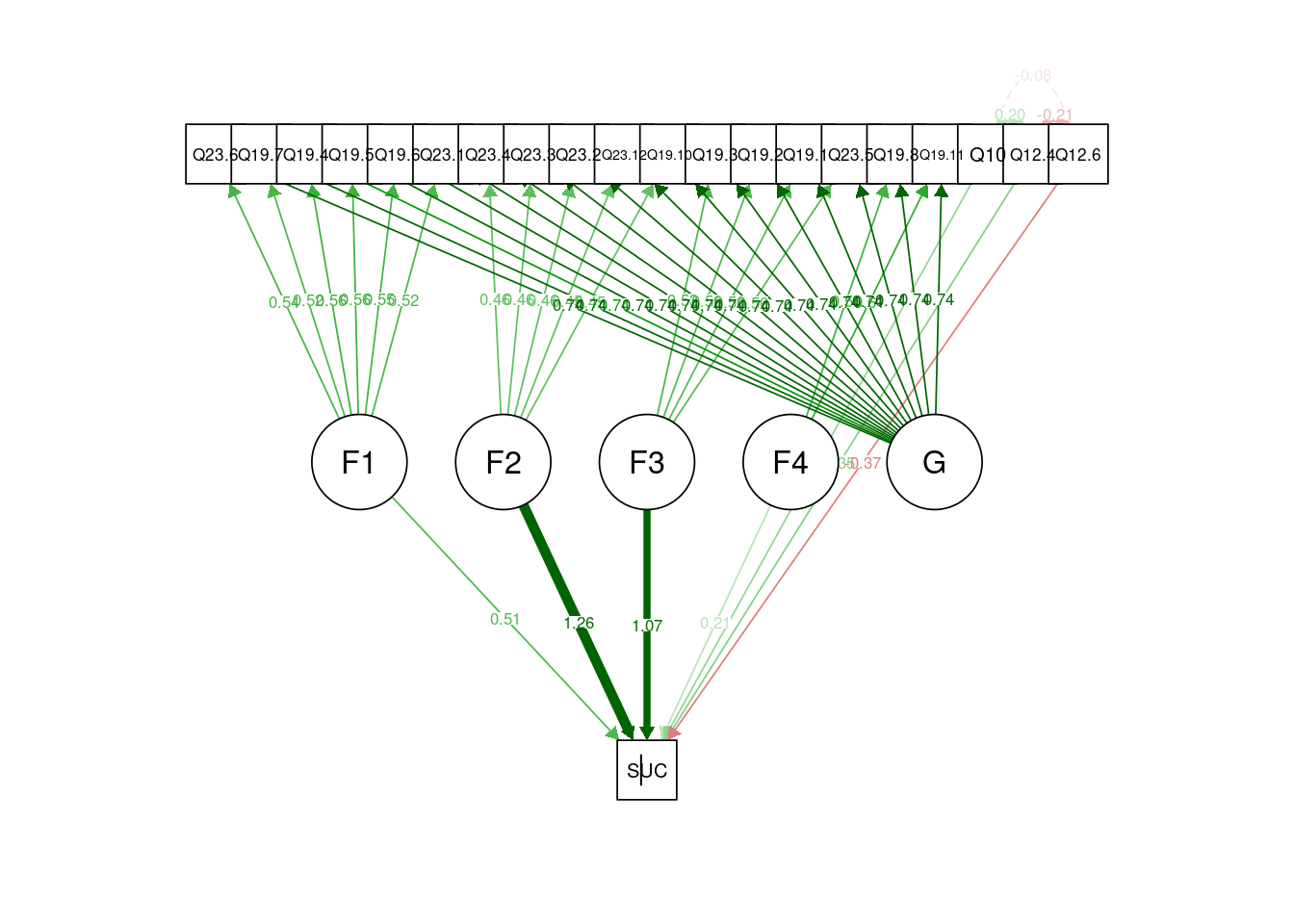
 ```

The final, 2-stage analysis

STAGE I

mod1 <- 'F1 =~ Q23.6 + Q19.7 + Q19.4 + Q19.5 + Q19.6 + Q23.1

F2 =~ Q23.4 + Q23.3 + Q23.2 + Q23.12 + Q19.10

F3 =~ Q19.3 + Q19.2 + Q19.1 + Q23.5

F4 =~ b*Q19.8 + b*Q19.11

G =~ a*Q23.6 + a*Q19.7 + a*Q19.4 + a*Q19.5 + a*Q19.6 + a*Q23.1 +

a*Q23.4 + a*Q23.3 + a*Q23.2 + a*Q23.12 + a*Q19.10 +

a*Q19.3 + a*Q19.2 + a*Q19.1 + a*Q23.5 +

a*Q19.8 + a*Q19.11'

fit_mod1 <- lavaan(mod1,data=data,auto.var=TRUE,std.ov=TRUE,std.lv=TRUE,missing="ml")

## Warning in lav_data_full(data = data, group = group, cluster = cluster, : lavaan WARNING: some cases are empty and will be ignored:

## 414

summary(fit_mod1,fit.measures=TRUE)

## lavaan 0.6-7 ended normally after 77 iterations

##

## Estimator ML

## Optimization method NLMINB

## Number of free parameters 51

## Number of equality constraints 17

##

## Used Total

## Number of observations 660 661

## Number of missing patterns 54

##

## Model Test User Model:

##

## Test statistic 388.444

## Degrees of freedom 136

## P-value (Chi-square) 0.000

##

## Model Test Baseline Model:

##

## Test statistic 18547.471

## Degrees of freedom 136

## P-value 0.000

##

## User Model versus Baseline Model:

##

## Comparative Fit Index (CFI) 0.986

## Tucker-Lewis Index (TLI) 0.986

##

## Loglikelihood and Information Criteria:

##

## Loglikelihood user model (H0) -6374.153

## Loglikelihood unrestricted model (H1) -6179.931

##

## Akaike (AIC) 12816.306

## Bayesian (BIC) 12969.042

## Sample-size adjusted Bayesian (BIC) 12861.091

##

## Root Mean Square Error of Approximation:

##

## RMSEA 0.053

## 90 Percent confidence interval - lower 0.047

## 90 Percent confidence interval - upper 0.059

## P-value RMSEA <= 0.05 0.204

##

## Standardized Root Mean Square Residual:

##

## SRMR 0.059

##

## Parameter Estimates:

##

## Standard errors Standard

## Information Observed

## Observed information based on Hessian

##

## Latent Variables:

## Estimate Std.Err z-value P(>|z|)

## F1 =~

## Q23.6 0.429 0.025 17.151 0.000

## Q19.7 0.422 0.025 16.860 0.000

## Q19.4 0.441 0.023 18.870 0.000

## Q19.5 0.470 0.025 19.082 0.000

## Q19.6 0.437 0.024 18.045 0.000

## Q23.1 0.426 0.026 16.209 0.000

## F2 =~

## Q23.4 0.598 0.024 24.695 0.000

## Q23.3 0.601 0.024 25.145 0.000

## Q23.2 0.559 0.024 23.377 0.000

## Q23.12 0.576 0.025 22.946 0.000

## Q19.10 0.534 0.024 22.330 0.000

## F3 =~

## Q19.3 0.534 0.024 22.083 0.000

## Q19.2 0.521 0.025 21.255 0.000

## Q19.1 0.501 0.025 20.054 0.000

## Q23.5 0.467 0.026 17.960 0.000

## F4 =~

## Q19.8 (b) 0.623 0.024 26.385 0.000

## Q19.11 (b) 0.623 0.024 26.385 0.000

## G =~

## Q23.6 (a) 0.797 0.025 32.315 0.000

## Q19.7 (a) 0.797 0.025 32.315 0.000

## Q19.4 (a) 0.797 0.025 32.315 0.000

## Q19.5 (a) 0.797 0.025 32.315 0.000

## Q19.6 (a) 0.797 0.025 32.315 0.000

## Q23.1 (a) 0.797 0.025 32.315 0.000

## Q23.4 (a) 0.797 0.025 32.315 0.000

## Q23.3 (a) 0.797 0.025 32.315 0.000

## Q23.2 (a) 0.797 0.025 32.315 0.000

## Q23.12 (a) 0.797 0.025 32.315 0.000

## Q19.10 (a) 0.797 0.025 32.315 0.000

## Q19.3 (a) 0.797 0.025 32.315 0.000

## Q19.2 (a) 0.797 0.025 32.315 0.000

## Q19.1 (a) 0.797 0.025 32.315 0.000

## Q23.5 (a) 0.797 0.025 32.315 0.000

## Q19.8 (a) 0.797 0.025 32.315 0.000

## Q19.11 (a) 0.797 0.025 32.315 0.000

##

## Intercepts:

## Estimate Std.Err z-value P(>|z|)

## .Q23.6 0.000

## .Q19.7 0.000

## .Q19.4 0.000

## .Q19.5 0.000

## .Q19.6 0.000

## .Q23.1 0.000

## .Q23.4 0.000

## .Q23.3 0.000

## .Q23.2 0.000

## .Q23.12 0.000

## .Q19.10 0.000

## .Q19.3 0.000

## .Q19.2 0.000

## .Q19.1 0.000

## .Q23.5 0.000

## .Q19.8 0.000

## .Q19.11 0.000

## F1 0.000

## F2 0.000

## F3 0.000

## F4 0.000

## G 0.000

##

## Variances:

## Estimate Std.Err z-value P(>|z|)

## .Q23.6 0.101 0.007 15.081 0.000

## .Q19.7 0.104 0.007 15.423 0.000

## .Q19.4 0.070 0.005 13.766 0.000

## .Q19.5 0.091 0.006 14.257 0.000

## .Q19.6 0.087 0.006 14.640 0.000

## .Q23.1 0.124 0.008 15.619 0.000

## .Q23.4 0.064 0.005 13.152 0.000

## .Q23.3 0.053 0.004 12.053 0.000

## .Q23.2 0.074 0.005 14.308 0.000

## .Q23.12 0.095 0.006 15.017 0.000

## .Q19.10 0.081 0.006 14.689 0.000

## .Q19.3 0.076 0.007 11.166 0.000

## .Q19.2 0.092 0.007 12.666 0.000

## .Q19.1 0.108 0.008 13.739 0.000

## .Q23.5 0.139 0.009 15.098 0.000

## .Q19.8 0.084 0.013 6.573 0.000

## .Q19.11 0.090 0.013 6.971 0.000

## F1 1.000

## F2 1.000

## F3 1.000

## F4 1.000

## G 1.000

Communalities (squared multiple correlations, SMC) for the observed variables.

par <- coef(fit_mod1)

npar <- length(par)

com <- 1-par[(npar-16):npar]

sort(com)

## Q23.5~~Q23.5 Q23.1~~Q23.1 Q19.1~~Q19.1 Q19.7~~Q19.7 Q23.6~~Q23.6

## 0.8611546 0.8760375 0.8921784 0.8961573 0.8991997

## Q23.12~~Q23.12 Q19.2~~Q19.2 Q19.5~~Q19.5 Q19.11~~Q19.11 Q19.6~~Q19.6

## 0.9045923 0.9082403 0.9092538 0.9097822 0.9130768

## Q19.8~~Q19.8 Q19.10~~Q19.10 Q19.3~~Q19.3 Q23.2~~Q23.2 Q19.4~~Q19.4

## 0.9159127 0.9186627 0.9242352 0.9261624 0.9296970

## Q23.4~~Q23.4 Q23.3~~Q23.3

## 0.9359113 0.9467742

Path diagram for the measurement model

semPaths(fit_mod1,"est",intercepts=FALSE,residuals=FALSE)


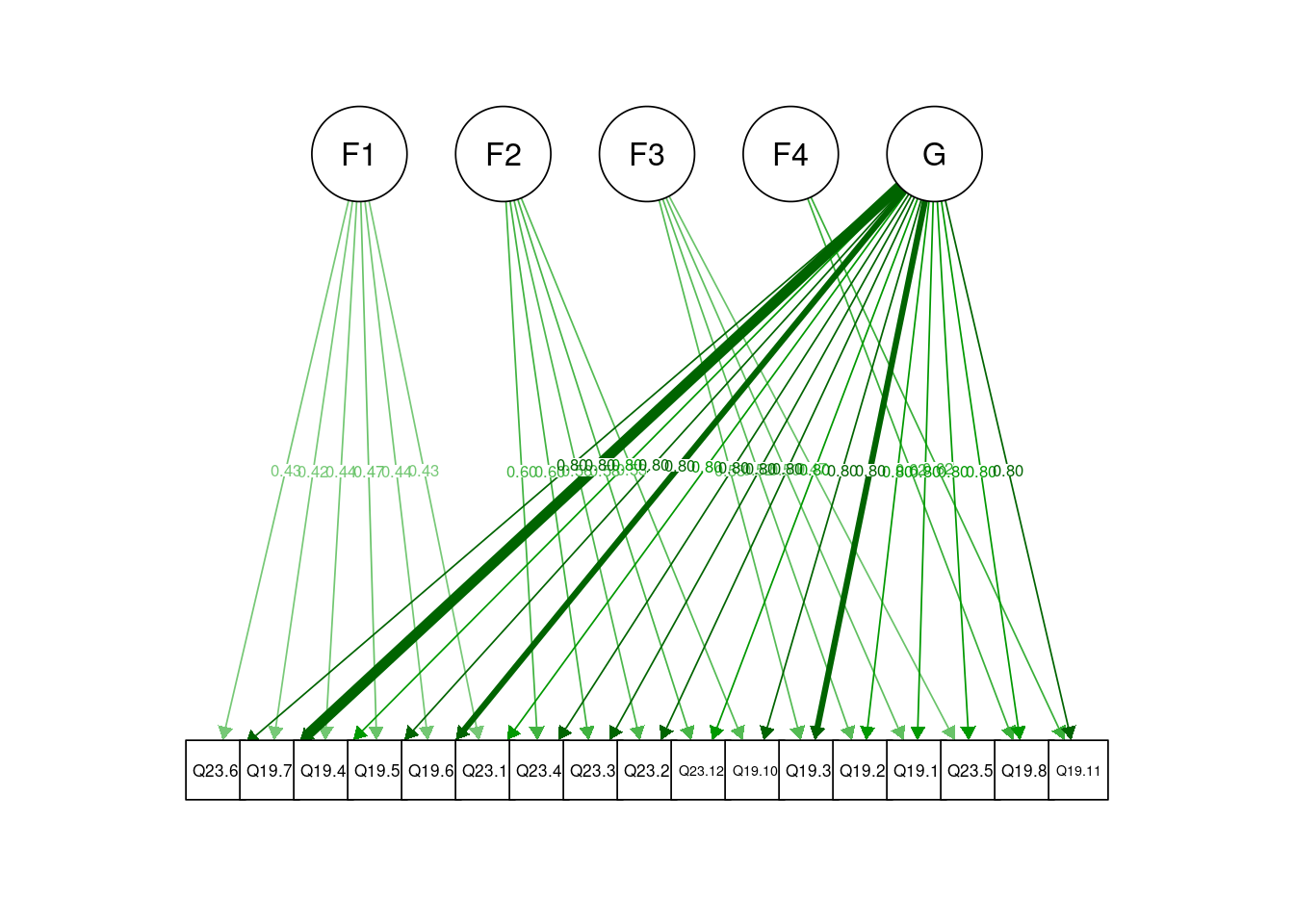


Looking at factor scores

FG <- lavPredict(fit_mod1)

op <- par(mfrow=c(2,2))

**for**(i **in** 1:4){

hist(FG[,i])

}


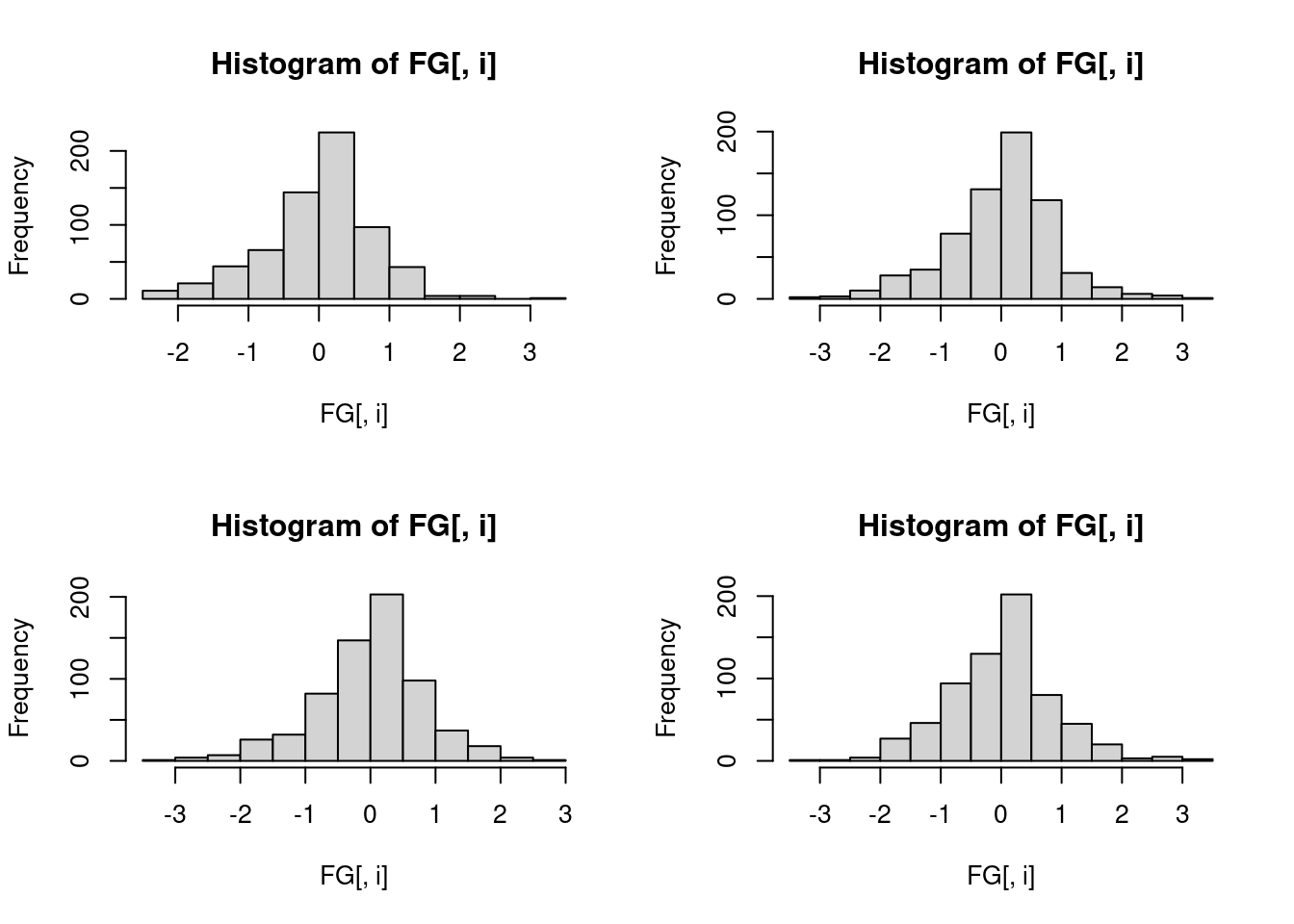


par(op)

hist(FG[,5],breaks=20,main="",xlab="Factor G")


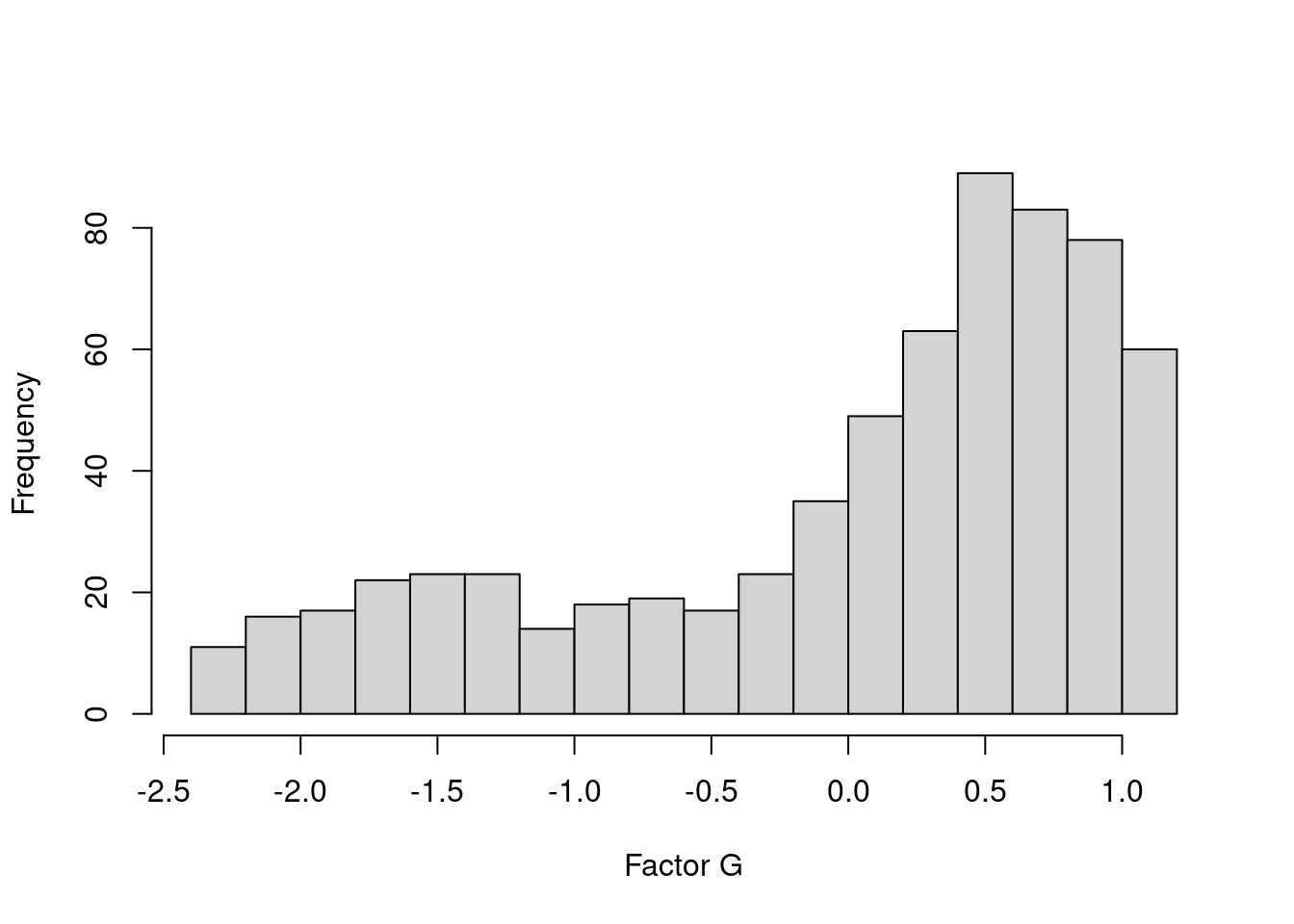


STAGE II

We first explain the success using only factors F1, F2, F3 and F4

data1 <- cbind(data,FG)

fit4 <- glm(SUCC~F1+F2+F3+F4,data=data1,family=binomial(link="probit"))

summary(fit4)

##

## Call:

## glm(formula = SUCC ~ F1 + F2 + F3 + F4, family = binomial(link = "probit"),

## data = data1)

##

## Deviance Residuals:

## Min 1Q Median 3Q Max

## -3.3452 -0.3003 0.2608 0.5520 2.7677

##

## Coefficients:

## Estimate Std. Error z value Pr(>|z|)

## (Intercept) 0.23209 0.06685 3.472 0.000516 ***

## F1 1.23582 0.14373 8.598 < 2e-16 ***

## F2 2.09179 0.15290 13.681 < 2e-16 ***

## F3 1.73006 0.14955 11.569 < 2e-16 ***

## F4 0.54967 0.12440 4.418 9.94e-06 ***

## ---

## Signif. codes: 0 '***' 0.001 '**' 0.01 '*' 0.05 '.' 0.1 ' ' 1

##

## (Dispersion parameter for binomial family taken to be 1)

##

## Null deviance: 884.50 on 656 degrees of freedom

## Residual deviance: 452.56 on 652 degrees of freedom

## (4 observations deleted due to missingness)

## AIC: 462.56

##

## Number of Fisher Scoring iterations: 6

nagelkerke(fit4,restrictNobs = TRUE)

## $Models

##

## Model: "glm, SUCC ~ F1 + F2 + F3 + F4, binomial(link = \"probit\"), data1"

## Null: "glm, SUCC ~ 1, binomial(link = \"probit\"), fit$model"

##

## $Pseudo.R.squared.for.model.vs.null

## Pseudo.R.squared

## McFadden 0.488339

## Cox and Snell (ML) 0.481821

## Nagelkerke (Cragg and Uhler) 0.651294

##

## $Likelihood.ratio.test

## Df.diff LogLik.diff Chisq p.value

## -4 -215.97 431.94 3.4905e-92

##

## $Number.of.observations

##

## Model: 657

## Null: 657

##

## $Messages

## [1] "Note: For models fit with REML, these statistics are based on refitting with ML"

##

## $Warnings

## [1] "None"

We check the result using logistic regression. The fit is slightly better.

fit4l <- glm(SUCC~F1+F2+F3+F4,data=data1,family=binomial)

summary(fit4l)

##

## Call:

## glm(formula = SUCC ~ F1 + F2 + F3 + F4, family = binomial, data = data1)

##

## Deviance Residuals:

## Min 1Q Median 3Q Max

## -3.2114 -0.2975 0.2633 0.5225 2.6938

##

## Coefficients:

## Estimate Std. Error z value Pr(>|z|)

## (Intercept) 0.4150 0.1219 3.406 0.000659 ***

## F1 2.2300 0.2740 8.138 4.01e-16 ***

## F2 3.7762 0.3053 12.368 < 2e-16 ***

## F3 3.1618 0.2948 10.726 < 2e-16 ***

## F4 0.8997 0.2326 3.868 0.000110 ***

## ---

## Signif. codes: 0 '***' 0.001 '**' 0.01 '*' 0.05 '.' 0.1 ' ' 1

##

## (Dispersion parameter for binomial family taken to be 1)

##

## Null deviance: 884.50 on 656 degrees of freedom

## Residual deviance: 445.75 on 652 degrees of freedom

## (4 observations deleted due to missingness)

## AIC: 455.75

##

## Number of Fisher Scoring iterations: 6

nagelkerke(fit4l,restrictNobs = TRUE)

## $Models

##

## Model: "glm, SUCC ~ F1 + F2 + F3 + F4, binomial, data1"

## Null: "glm, SUCC ~ 1, binomial, fit$model"

##

## $Pseudo.R.squared.for.model.vs.null

## Pseudo.R.squared

## McFadden 0.496040

## Cox and Snell (ML) 0.487166

## Nagelkerke (Cragg and Uhler) 0.658519

##

## $Likelihood.ratio.test

## Df.diff LogLik.diff Chisq p.value

## -4 -219.37 438.75 1.1763e-93

##

## $Number.of.observations

##

## Model: 657

## Null: 657

##

## $Messages

## [1] "Note: For models fit with REML, these statistics are based on refitting with ML"

##

## $Warnings

## [1] "None"

Next, we fit a model where the predictors are the factors + Q10, Q12.4, Q12.6

(working ability before rehabilitation, the aim of improving professional skills, the aim of receiving a disability pension)

fit5 <- glm(SUCC~F1+F2+F3+F4+Q10 + Q12.4 + Q12.6,data=data1,family=binomial)

summary(fit5)

##

## Call:

## glm(formula = SUCC ~ F1 + F2 + F3 + F4 + Q10 + Q12.4 + Q12.6,

## family = binomial, data = data1)

##

## Deviance Residuals:

## Min 1Q Median 3Q Max

## -3.1090 -0.2483 0.2085 0.4617 2.7654

##

## Coefficients:

## Estimate Std. Error z value Pr(>|z|)

## (Intercept) -1.37307 0.60435 -2.272 0.023089 *

## F1 2.00532 0.29712 6.749 1.49e-11 ***

## F2 3.55624 0.33186 10.716 < 2e-16 ***

## F3 3.05972 0.32058 9.544 < 2e-16 ***

## F4 0.76934 0.25738 2.989 0.002798 **

## Q10 0.23974 0.09820 2.441 0.014630 *

## Q12.4 0.54878 0.12046 4.556 5.22e-06 ***

## Q12.6 -0.32966 0.09054 -3.641 0.000271 ***

## ---

## Signif. codes: 0 '***' 0.001 '**' 0.01 '*' 0.05 '.' 0.1 ' ' 1

##

## (Dispersion parameter for binomial family taken to be 1)

##

## Null deviance: 860.82 on 640 degrees of freedom

## Residual deviance: 382.43 on 633 degrees of freedom

## (20 observations deleted due to missingness)

## AIC: 398.43

##

## Number of Fisher Scoring iterations: 6

nagelkerke(fit5,restrictNobs=TRUE)

## $Models

##

## Model: "glm, SUCC ~ F1 + F2 + F3 + F4 + Q10 + Q12.4 + Q12.6, binomial, data1"

## Null: "glm, SUCC ~ 1, binomial, fit$model"

##

## $Pseudo.R.squared.for.model.vs.null

## Pseudo.R.squared

## McFadden 0.555741

## Cox and Snell (ML) 0.525892

## Nagelkerke (Cragg and Uhler) 0.711704

##

## $Likelihood.ratio.test

## Df.diff LogLik.diff Chisq p.value

## -7 -239.2 478.39 3.5359e-99

##

## $Number.of.observations

##

## Model: 641

## Null: 641

##

## $Messages

## [1] "Note: For models fit with REML, these statistics are based on refitting with ML"

##

## $Warnings

## [1] "None"

Next, we fit a model where the predictors are the factors + Q1.1 , Q10, Q11, Q12.4, Q12.6 (age, working ability before rehabilitation, motivation, the aim of improving professional skills, the aim of receiving a disability pension)

fit6 <- glm(SUCC~F1+F2+F3+F4+ Q1.1 + Q10 + Q11 + Q12.4 +Q12.6,data=data1,family=binomial)

summary(fit6)

##

## Call:

## glm(formula = SUCC ~ F1 + F2 + F3 + F4 + Q1.1 + Q10 + Q11 + Q12.4 +

## Q12.6, family = binomial, data = data1)

##

## Deviance Residuals:

## Min 1Q Median 3Q Max

## -3.2650 -0.2387 0.2002 0.4347 2.9702

##

## Coefficients:

## Estimate Std. Error z value Pr(>|z|)

## (Intercept) -0.60526 1.35187 -0.448 0.654356

## F1 1.91294 0.30233 6.327 2.49e-10 ***

## F2 3.39944 0.33047 10.287 < 2e-16 ***

## F3 2.99954 0.32087 9.348 < 2e-16 ***

## F4 0.71248 0.25658 2.777 0.005490 **

## Q1.1 -0.03926 0.01736 -2.262 0.023718 *

## Q10 0.22364 0.10057 2.224 0.026164 *

## Q11 0.38425 0.18021 2.132 0.032986 *

## Q12.4 0.40743 0.13147 3.099 0.001941 **

## Q12.6 -0.32178 0.09363 -3.437 0.000589 ***

## ---

## Signif. codes: 0 '***' 0.001 '**' 0.01 '*' 0.05 '.' 0.1 ' ' 1

##

## (Dispersion parameter for binomial family taken to be 1)

##

## Null deviance: 857.96 on 638 degrees of freedom

## Residual deviance: 371.89 on 629 degrees of freedom

## (22 observations deleted due to missingness)

## AIC: 391.89

##

## Number of Fisher Scoring iterations: 6

nagelkerke(fit6,restrictNobs=TRUE)

## $Models

##

## Model: "glm, SUCC ~ F1 + F2 + F3 + F4 + Q1.1 + Q10 + Q11 + Q12.4 + Q12.6, binomial, data1"

## Null: "glm, SUCC ~ 1, binomial, fit$model"

##

## $Pseudo.R.squared.for.model.vs.null

## Pseudo.R.squared

## McFadden 0.566539

## Cox and Snell (ML) 0.532645

## Nagelkerke (Cragg and Uhler) 0.720913

##

## $Likelihood.ratio.test

## Df.diff LogLik.diff Chisq p.value

## -9 -243.03 486.07 5.5282e-99

##

## $Number.of.observations

##

## Model: 639

## Null: 639

##

## $Messages

## [1] "Note: For models fit with REML, these statistics are based on refitting with ML"

##

## $Warnings

## [1] "None"

We still fit a model predicting return to work

fit7 <- glm(RTW~F1+F2+F3+F4+Q1.1+Q10 +Q11 + Q12.4 + Q12.6,data=data1,family=binomial)

summary(fit7)

##

## Call:

## glm(formula = RTW ~ F1 + F2 + F3 + F4 + Q1.1 + Q10 + Q11 + Q12.4 +

## Q12.6, family = binomial, data = data1)

##

## Deviance Residuals:

## Min 1Q Median 3Q Max

## -1.9681 -0.8236 -0.4041 0.9504 2.5882

##

## Coefficients:

## Estimate Std. Error z value Pr(>|z|)

## (Intercept) 0.44591 0.95738 0.466 0.64139

## F1 1.14943 0.23689 4.852 1.22e-06 ***

## F2 1.61056 0.24032 6.702 2.06e-11 ***

## F3 1.21714 0.23607 5.156 2.53e-07 ***

## F4 0.56700 0.20089 2.822 0.00477 **

## Q1.1 -0.01841 0.01160 -1.588 0.11235

## Q10 0.09185 0.07334 1.252 0.21041

## Q11 0.08545 0.14301 0.598 0.55014

## Q12.4 -0.10011 0.10033 -0.998 0.31841

## Q12.6 -0.34636 0.06840 -5.063 4.12e-07 ***

## ---

## Signif. codes: 0 '***' 0.001 '**' 0.01 '*' 0.05 '.' 0.1 ' ' 1

##

## (Dispersion parameter for binomial family taken to be 1)

##

## Null deviance: 785.03 on 641 degrees of freedom

## Residual deviance: 635.03 on 632 degrees of freedom

## (19 observations deleted due to missingness)

## AIC: 655.03

##

## Number of Fisher Scoring iterations: 5

nagelkerke(fit7,restrictNobs=TRUE)

## $Models

##

## Model: "glm, RTW ~ F1 + F2 + F3 + F4 + Q1.1 + Q10 + Q11 + Q12.4 + Q12.6, binomial, data1"

## Null: "glm, RTW ~ 1, binomial, fit$model"

##

## $Pseudo.R.squared.for.model.vs.null

## Pseudo.R.squared

## McFadden 0.191066

## Cox and Snell (ML) 0.208347

## Nagelkerke (Cragg and Uhler) 0.295281

##

## $Likelihood.ratio.test

## Df.diff LogLik.diff Chisq p.value

## -9 -74.996 149.99 8.8537e-28

##

## $Number.of.observations

##

## Model: 642

## Null: 642

##

## $Messages

## [1] "Note: For models fit with REML, these statistics are based on refitting with ML"

##

## $Warnings

## [1] "None"

We still fit a model predicting self-asessed ability to work

fit8 <- glm(SRH~F1+F2+F3+F4+ Q1.1 + Q10 + Q11 + Q12.4 + Q12.6,data=data1,family=binomial)

summary(fit8)

##

## Call:

## glm(formula = SRH ~ F1 + F2 + F3 + F4 + Q1.1 + Q10 + Q11 + Q12.4 +

## Q12.6, family = binomial, data = data1)

##

## Deviance Residuals:

## Min 1Q Median 3Q Max

## -3.1816 -0.2563 0.2087 0.4737 3.1640

##

## Coefficients:

## Estimate Std. Error z value Pr(>|z|)

## (Intercept) -1.49741 1.32223 -1.132 0.257429

## F1 2.23658 0.31659 7.065 1.61e-12 ***

## F2 3.50754 0.34225 10.249 < 2e-16 ***

## F3 3.24303 0.34372 9.435 < 2e-16 ***

## F4 1.15971 0.26460 4.383 1.17e-05 ***

## Q1.1 -0.03103 0.01669 -1.859 0.062999 .

## Q10 0.27097 0.09764 2.775 0.005517 **

## Q11 0.39570 0.17854 2.216 0.026668 *

## Q12.4 0.42309 0.12933 3.271 0.001070 **

## Q12.6 -0.33728 0.09072 -3.718 0.000201 ***

## ---

## Signif. codes: 0 '***' 0.001 '**' 0.01 '*' 0.05 '.' 0.1 ' ' 1

##

## (Dispersion parameter for binomial family taken to be 1)

##

## Null deviance: 868.72 on 637 degrees of freedom

## Residual deviance: 390.10 on 628 degrees of freedom

## (23 observations deleted due to missingness)

## AIC: 410.1

##

## Number of Fisher Scoring iterations: 6

nagelkerke(fit8,restrictNobs=TRUE)

## $Models

##

## Model: "glm, SRH ~ F1 + F2 + F3 + F4 + Q1.1 + Q10 + Q11 + Q12.4 + Q12.6, binomial, data1"

## Null: "glm, SRH ~ 1, binomial, fit$model"

##

## $Pseudo.R.squared.for.model.vs.null

## Pseudo.R.squared

## McFadden 0.550950

## Cox and Snell (ML) 0.527722

## Nagelkerke (Cragg and Uhler) 0.709536

##

## $Likelihood.ratio.test

## Df.diff LogLik.diff Chisq p.value

## -9 -239.31 478.62 2.1689e-97

##

## $Number.of.observations

##

## Model: 638

## Null: 638

##

## $Messages

## [1] "Note: For models fit with REML, these statistics are based on refitting with ML"

##

## $Warnings

## [1] "None"
